# The potential of olfaction loss to induce cognitive impairment and anxiety behavior in mice via the microbiota-gut-brain axis

**DOI:** 10.3389/fmicb.2025.1595742

**Published:** 2025-07-02

**Authors:** Xiangyu Zhao, Chao Xue, Yiming Wang, Xiwei Liu, Ran Li, Xianfeng Yi

**Affiliations:** School of Life Sciences, Qufu Normal University, Qufu, China

**Keywords:** olfactory loss, cognitive impairment, anxiety behavior, gut microbiota, microbiota-gut-brain axis

## Abstract

**Introduction:**

Olfactory dysfunction and cognition decline are frequently observed; however, very little is known about whether olfactory disorders trigger cognitive impairment.

**Methods:**

Here, we induced olfactory loss in mice and investigated whether and how olfactory loss induces cognitive impairment and anxiety behavior.

**Results:**

Olfactory loss not only causes a significant decrease in food intake and body weight and an increase in O_2_ consumption but also induces cognitive impairment and anxiety behavior. Olfactory loss-induced alteration of the gut microbiota is associated with subsequent changes in cecal short-chain fatty acids and serum neurotransmitter levels. Hippocampus proteome and fecal microbial transplantation provide further support for the mechanisms by which olfactory loss triggers cognitive impairment and anxiety behavior via the microbiota-gut-brain axis.

**Discussion:**

Our study is expected to provide some evidence for olfactory dysfunction in triggering cognitive impairment through the microbiota-gut-brain axis.

## 1 Introduction

The sense of smell is a significant physiological function, contributing to danger recognition, social interactions, and dietary experiences (Stevenson, 2010). Olfactory health warrants attention, as exposure to pollutants like fine particulate matter (PM2.5) and traffic-related emissions, has been tentatively linked to diminished olfactory capacity (Doty, 2021; Guadalupe-Fernandez et al., 2021; Ekström et al., 2022). Additionally, during the COVID-19 pandemic, temporary smell loss emerged as a commonly reported symptom (Gerkin et al., 2021; Pieniak et al., 2022).

Connections between olfaction and broader physiological processes, including aspects of cognition, have been explored. For instance, olfactory decline has been observed alongside cognitive changes in neurodegenerative disorders that remain incompletely understood (Dahmani et al., 2018). A proposed hypothesis suggests that agents entering the brain via the olfactory pathway might influence neurodegenerative processes. Some studies note olfactory impairment and pathological changes in olfactory regions, alongside evidence that certain environmental agents can access the brain through olfactory pathways (Doty, 2008). For example, intravenous administration of 1-methyl 4-phenyl 1,2,3,6-tetrahydropyridine (MPTP), a neurotoxin linked to parkinsonism, did not significantly impair olfaction in humans (Doty et al., 1992). In contrast, intranasal MPTP exposure in rats led to gradual olfactory, cognitive, and motor deficits (Prediger et al., 2006). The olfactory decline has also been associated with cognitive changes in aging and other conditions (Vance et al., 2024), with studies reporting correlations between smell impairment and cognitive differences in middle-aged and older adults (Devanand, 2016; Adams et al., 2018), as well as tentative links between olfactory dysfunction and transitions from normal cognition to mild cognitive impairment (Yan et al., 2022).

Some research indicates that olfactory enhancement may be correlated with cognitive improvements. For instance, olfactory training has been linked to modest cognitive benefits in certain studies (Vance et al., 2024), while odor enrichment in mice was shown to increase hippocampal neuron counts (Rusznak et al., 2018), and prolonged odor exposure reduced tau phosphorylation in specific brain regions of rats (Liao et al., 2012). Although many studies emphasize cognitive improvements associated with enhanced olfaction, fewer investigations focus on how olfactory decline might contribute to cognitive changes. In Alzheimer's disease (AD), for example, olfactory and cognitive symptoms frequently co-occur; however, some evidence suggests that olfactory changes may precede noticeable cognitive decline (Bathini et al., 2019). Further exploration of this relationship could yield valuable insights into the pathophysiological mechanisms underlying these conditions.

The mechanisms linking olfaction and cognition remain unclear. One theory posits that olfactory pathways interact with memory-related brain regions like the hippocampus and entorhinal cortex, damage to which may contribute to cognitive changes (Vance et al., 2024). Additionally, emerging evidence highlights potential indirect pathways, such as gut microbiota-brain interactions. Gut microbes influence neurotransmitter production (e.g., GABA, serotonin) and may modulate brain function (Forsythe et al., 2014; Lyte, 2014). Studies suggest microbiota transplants from cognitively impaired donors to rodents can alter recipient behavior (Li Y. L. et al., 2020; Wang et al., 2021), and probiotics have been linked to improved memory in mice (Ohland et al., 2013).

Olfactory dysfunction may indirectly affect cognition through dietary changes. Smell loss can alter appetite and food choices (Yeomans, 2006; Connor et al., 2018), potentially influencing metabolism and weight (Hintze et al., 2019; Fjaeldstad and Smith, 2022). Diet shapes gut microbiota composition (Maurer et al., 2024; Wang et al., 2025), which in turn may interact with brain function via the gut-brain axis (Li Y. L. et al., 2020; Wang et al., 2021). Intriguingly, microbiota changes might reciprocally affect olfaction and cognition (Naudon et al., 2020; Zhao et al., 2024). This interplay raises the possibility that olfactory dysfunction could influence cognitive health through gut microbial pathways, though further investigation is needed.

Here, this study investigated whether olfactory loss induces cognitive impairment and anxiety via the gut microbiota-brain axis in C57BL/6 mice. Olfactory dysfunction was chemically induced using intranasal Triton X-100, selectively targeting the olfactory epithelium (Iqbal and Byrd-Jacobs, 2010; Yi et al., 2021). The research aimed to test four predictions: (1) Olfactory loss would cause cognitive deficits and anxiety-like behaviors; (2) Gut microbiota alterations would mediate these effects; (3) Metabolites (neurotransmitters and short-chain fatty acids) would regulate cognition via this axis; and (4) Hippocampal protein pathways would underlie impairment. Methodology included: verifying olfactory loss (buried food test, histology, olfactory bulb proteomics); monitoring physiological changes (food intake, weight, metabolism); analyzing gut microbiota (16S rDNA sequencing) and metabolites; assessing behavior (cognitive/anxiety tests); and validating mechanisms via hippocampal proteomics and fecal microbiota transplantation (FMT). Our results will provide some evidence that olfactory loss triggers cognitive impairment through microbiota-gut-brain axis disruption, involving microbial shifts, metabolite changes, and hippocampal pathway alterations. This work is expected to establish a theoretical link between olfaction and cognition, suggesting potential therapeutic strategies for neurodegenerative disorders where olfactory and cognitive deficits co-occur.

## 2 Materials and methods

### 2.1 Animals and handling

Eight-week-old C57BL/6J mice (Jinan Pengyue Experimental Animal Breeding Co., China) were housed individually in polystyrene plastic cages (37 cm × 27 cm × 17 cm) where they had free access to water and standard rat chow. After 1 week of acclimatization, the mice were randomly divided into the control group (*n* = 20 10 males and 10 females) and the anosmic group (*n* = 20 10 males and 10 females). For the control group, each individual received bilateral nasal instillation of 50 μl 0.9% saline solution two times per day for 7 days. Similarly, each individual of the anosmic group received a bilateral nasal instillation of 50 μl 0.7% Triton X-100 dissolved in 0.9% saline in the same procedure. In our previous study, *Leopoldamys edwardsi* showed no significant change in caching behavior when processed with the same methodology (Yi et al., 2021). Chronic Triton X-100 treatment caused fish to lose responses to social odorants but retain responses to feeding-related odorants, though sensitivity to social odorants eventually returned (Paskin and Byrd-Jacobs, 2012). Additionally, evidence shows that Triton X-100 reversibly damages the olfactory epithelium, leading to degeneration and regeneration within weeks post-treatment (Iqbal and Byrd-Jacobs, 2010). Thus, despite a lack of direct evidence, we believe it is unlikely that this treatment's side effects significantly impact the gut microbiota or behaviors unrelated to olfaction. Nasal gavage was administrated slowly to avoid causing aspiration pneumonia and entering the esophagus. During and after administration, the body weight and food intake were measured every 2 days for 20 and 18 days, respectively. To measure daily food intake, C57BL/6J mice were provided with a fixed amount of food. After 24 h, the remaining food was collected and weighed to calculate consumption. For body weight measurements, mice were fasted for 3 h before testing and then weighed using a standard balance. We also performed the buried food-seeking test three times to see if the administration of Triton X-100 successfully induced olfaction loss. After that, individuals of the two groups were subjected to behavioral tests including cognition ability on the Barnes maze and anxiety-like behavior in the open-field arena.

### 2.2 Buried food-seeking test

Twenty individuals from each group were used in the buried food-seeking experiment. To test whether intranasal irrigation with Triton X-100 successfully induced olfaction loss in mice, a buried food-seeking test was conducted on all individuals of the control and anosmic groups, respectively. We buried one pellet of rat chow 1 cm deep in the fine sand contained in a wooden box (1.5 m × 0.5 m × 0.5 m). Then, we introduced one individual of mice into the box and allowed it to search freely for the buried pellet for 5 min. The time spent by each mouse to excavate the buried food pellet was recorded and was compared to test if olfaction was lost. However, the test was terminated if the mice failed to locate the food pellet within 5 min.

### 2.3 O_2_ consumption

Six individuals were randomly selected from each group for the experiment. The metabolic rate of the mice was measured under ambient temperature using an open-flow respirometry system (Q-Box RP2LP, Qubit, Canada), following the manufacturer's instructions. Mice were fasted for 3 h before testing to minimize the potential influence of feeding on body weight. Testing was conducted between 2:00 and 5:00 p.m. O_2_ consumption was recorded after mice had acclimated in the chamber for at least 20 min.

### 2.4 Barnes maze test

Twenty individuals from the control and anosmic groups were tested on the Barnes maze, respectively. The Barnes maze consists of a circular platform 140 cm above the ground and 91 cm in diameter. The platform has 20 equally spaced circular holes (5 cm × 5 cm). Only one of these holes was connected to a black box (the target hole). The Any-maze 7.0 Animal Behavior Video Analysis System (Stoelting, USA), was used to monitor the mice above the center of the maze. Mice were placed in an opaque box in the middle of the maze for 5 s before each training session. After removing the box, the mice were allowed to roam freely on the platform to locate the target box. The training lasted for 4 min and ended when the mice entered the target box with the whole body for 30 s. If the mice failed to locate the target box within 4 min, they were manually placed inside for 30 s. To minimize the chance that mice would rely on odors to determine the location of the target box, the maze was rotated randomly between each experiment. The orientation of the target box was always fixed, and the maze was cleaned with 75% alcohol after each training session to avoid odor interference. Each mouse of the two groups was trained once a day for 6 consecutive days. On day 7, the spatial cognition capacity of each mouse was tested using the same procedure.

### 2.5 Open-field tests

The open-field arena consists of a 45 cm × 45 cm × 40 cm (L × W × H) uncovered transparent partition with a black bottom, in which a 9 cm width of the edge area is named the marginal area, and the rest of the area called the center area. Between 2:00 and 5:00 p.m., mice were individually introduced into the open field arena and monitored for 5 min. The Any-maze 7.0 Animal Behavior Video Analysis System (Stoelting Co., USA) was placed on top of the open field to record the movement trajectories of the mice and the time spent in the center. More time spent in the edges of the chamber was interpreted as anxiety-like behavior. To avoid odor interference, the apparatus was cleaned with 75% alcohol between every two bouts of tests. Nineteen individuals from each group were successfully tested in the open-field experiment.

### 2.6 Fecal microbiota transplantation (FMT)

Eight-week-old C57 mice used for the FMT experiments were purchased from Jinan Pengyue Experimental Animal Breeding Co. and maintained under the same conditions described in Section 2.1 (Animals and Handling). One week after acclimatization, the recipient mice were randomly divided into two groups, A-FMT (*n* = 10) and C-FMT (*n* = 10), to receive fecal microbiota collected from the control and anosmic groups of mice, respectively. Fresh mouse feces from the control and anosmic groups were collected in the sterile EP tubes, respectively, and then dissolved in 0.9 % sterile saline to obtain a suspended solution of 0.1 g/ml of fecal bacteria. The solution was centrifuged at 500 r/min at 37°C for 10 min and the supernatant was collected for gavage. Each recipient mouse received 300 μl fecal suspension of donors every day for 7 consecutive days. Fresh feces samples from the control and anosmic groups were collected daily for gavage to ensure the freshness of the fecal suspension. During the gavage period, mice were guaranteed free access to water and standard rat chow *ad libitum*. Four weeks after FMT, the Barnes maze was used to test the difference in cognitive ability between the recipients using the same procedure as above. Upon completion of the Barnes maze experiment, fecal samples from mice were collected for gut microbiota analysis. For sample collection, each mouse was individually placed in a pre-sterilized cage equipped with sterile paper lining. The animals were retained in the cages for no longer than 10 min, during which freshly defecated feces were promptly collected from the sterile paper (Zhou et al., 2022). The collected samples were immediately submerged in liquid nitrogen and subsequently stored at −80°C until further analysis. Five individuals of each group were randomly selected for gut microbiota analysis.

### 2.7 Sample collection of mice

After the behavioral tests and the fecal microbiota transplantation (FMT) experiment, seven individuals of C57 mice from each group (anosmic group and control group) were randomly selected and anesthetized. For serum metabolite analysis, blood samples were extracted via cardiac puncture and centrifuged to collect serum into the sterile EP tubes, snap-frozen in liquid nitrogen, and stored at −80°C. Subsequently, the animals were euthanized by cervical dislocation following isoflurane anesthesia (Yi and Cha, 2022) and immediately dissected on ice to collect the bilateral hippocampi, the olfactory bulbs, and the cecal contents as quickly as possible. For subsequent hippocampal and olfactory bulb proteomic assays, gut microbiota testing, and short-chain fatty acid measurement, all samples were immediately frozen in liquid nitrogen and stored at −80°C.

### 2.8 Hematoxylin-eosin (HE) staining of the olfactory epithelium

Considering the extensive body of literature documenting the detrimental effects of Triton X-100 on the olfactory epithelium, as well as our confirmation of olfactory dysfunction in the anosmic group using the Buried Food-Seeking Test, two individuals from each group were randomly chosen for HE staining experiments, and six photographs were taken randomly in each of the anterior, middle, and terminal segments of the nasal cavity for cell number analysis. Intact nasal cavities were isolated and embedded in paraffin blocks, and paraffin sections were prepared routinely. Xylene was used to dewax the paraffinized nasal cavity sections, gradient ethanol was used to dehydrate them, distilled water was used to wash them, and hematoxylin was used to stain them. Finally, tap water was used to slowly rinse the paraffinized sections. Differential staining was carried out using 1% hydrochloric acid–alcohol solution for 30 s, and the sections were slowly rinsed with tap water for at least 5 min, followed by staining in 1% eosin Y solution for 30 s and slow rinsing with tap water for at least 15 min. The sections were dehydrated with an ethanol gradient, cleared with xylene, and then sealed with neutral resin. The morphological features of the olfactory epithelium were observed under a light microscope.

### 2.9 Analysis of the gut microbiota of mice

Seven individuals each in the anosmic and control group, and five individuals each in the A-FMT and C-FMT group were used for the gut microbiota 16S rDNA assay. The cecal contents (anosmic group and control group) and fecal samples (A-FMT and C-FMT) were transported on dry ice to OE Biotech Shanghai for testing following the manufacturer's instructions. Isolating bacterial DNA from feces and cecum contents was accomplished using a MagPure Soil DNA LQ kit (Magen, Guangdong, China). We determined the DNA concentration using a NanoDrop 2000 spectrophotometer (Thermo Fisher Scientific, Waltham, MA, USA) and examined the DNA integrity via agarose gel electrophoresis. A universal primer pair (343F: 5′- TACGGRAGGCAGAG-3′; 798 r:5′-AGGGTATCTAATCCT3′) was used. The reverse primer contains the sample barcode, and two primers are connected to the Illumina sequencing adapter.

#### 2.9.1 Library construction and sequencing

Electrophoresis on agarose gels was used to visualize amplicon quality. A second round of PCR was performed after AMPure XP beads (Agencourt) were purified from the PCR products. The final amplicon was quantified using a Qubit dsDNA Assay Kit (Thermo Fisher Scientific, USA) after purification with AMPure XP beads. Sequencing was performed on an Illumina NovaSeq 6000 platform with 250 bp paired-end reads (Illumina Inc., San Diego, CA; OE Biotech Company, Shanghai, China).

#### 2.9.2 Bioinformatic analysis

OE Biotech Co., Ltd. (Shanghai, China) performed the sequencing and data processing. The raw sequencing data were in FASTQ format. The paired-end reads were then preprocessed using Cutadapt software to detect and remove the adapters. Low-quality sequences were filtered from paired-end reads, denoised, merged, and detected, and chimeric reads were removed using DADA2 with the default parameters of QIIME2. The software then outputs the representative reads of each ASV and its abundance table. The representative reads were selected using the QIIME2 package. A q2-feature classifier with default parameters was used to annotate and blast representative reads against the Unite database. QIIME2 software was used for alpha and beta diversity analysis. Using the alpha diversity indices, which include the Chao1 index and Shannon index, the microbial diversity of the samples was estimated. To estimate beta diversity, an unweighted UniFrac distance matrix generated by R was used in the unweighted UniFrac principal coordinates analysis (PCoA). Then, the R package was used to analyze the significant differences between different groups using ANOVA, the Kruskal–Wallis/T test, and the Wilcoxon statistical test. The linear discriminant analysis effect size (LEfSe) method was used to compare the taxonomy abundance spectra.

### 2.10 GC-MS/MS targeted serum metabolomics analysis

The targeted serum metabolomics analysis encompassed six individuals in each group. Regarding the targeted serum metabolomics analysis, both an experimental platform and assistance were offered by Shanghai Lu-Ming Biotech Company Limited in Shanghai, China. The serum samples kept at −80°C were retrieved and thawed at room temperature. Appropriate samples were taken and combined with 300 μL of a methanol: acetonitrile solution (v/v = 2:1, containing 0.1% formic acid, 0.1 mM/L BHT, and internal standards succinic acid-2,2,3,3-d4 and Lyso PC17:0). Vortex for 1 min, ultrasonic for 10 min, stand at −20°C; Centrifuge for 10 min (4°C, 12000 r), take 400 μL supernatant and dry; Redissolved with 200 μL water (containing internal standard L-2-chlorophenylalanine), swirled for 30 s, and ultrasounded in ice water bath for 5 min; Centrifuge for 5 min (4°C, 13000 rpm), take 150 μL supernatant and transfer it to the brown LC injection bottle, and stored it at −80°C until machine analysis.

The quantitative detection of targeted amino acid metabolites was performed using UPLC-ESI-MS/MS as the analytical method. Sample size: 5 μL; Flow rate: 0.3 mL/min; Mobile phase: A (0.1% formic-aqueous solution), B (0.1% formic-methanol); Gradient Elution Procedures: 0 min A/B (99:1, V/V), 1 min A/B (99:1, V/V), 6min A/B (5:95, V/V), 7 min A/B (5:95, V/V), 7.01 min A/B (99:1, V/V), 8min A/B (99:1, V/V) V/V).

Samples were analyzed by a highly sensitive mass spectrometer (AB Sciex Qtrap 6500+) and a high-performance liquid chromatograph (AB ExionLC). The chromatographic column was ACQUITY UPLC HSS PFP (100 mm × 2.1 mm, 1.8 μm). The specific analysis conditions and methods of the experiment are as follows: gas curtain gas: 30 (psi); collision-activated dissociation (CAD) parameters: medium; positive ion spray voltage: 5500 V; negative ion spray voltage: −4500 V; ion source temperature: 450°C; column temperature: 40°C; spray gas (Gas1): 50 (psi); auxiliary heating gas (Gas2): 50 (psi).

The analysis of metabolites was conducted through the multiple reaction monitoring (MRM) mode in triple quadrupole mass spectrometry. The quadrupole in MRM mode first selects the precursor ions of the target substance, eliminating ions of different molecular weights to minimize interference. Following ionization in the collision chamber, the precursor ion dissociates to produce various fragment ions. A specific fragment ion is then selected using a triple quadrupole filter to remove the interference from non-target ions, resulting in improved accuracy and better repeatability in quantification. Mass spectrometry data from various samples were collected, and the peak areas of all chromatographic peaks were integrated, with corrections applied to the chromatographic peaks of the same substance across different samples.

### 2.11 Measurement of short-chain fatty acids

Seven individuals per group were used for the short-chain fatty acids analysis. The cecal samples were transported on dry ice to Shanghai Lumineers for analysis of short-chain fatty acids. Following the manufacturer's instructions, the appropriate sample was weighed, and 300 liters of 50% acetonitrile-water solution (v/v) containing the internal standards (Pentanoic acid [2H9] and Hexanoic acid [2H11]) was placed in the reaction vessel. Samples were ground for 3 min (precooled to −20°C), then ultrasonically cooled for 10 min, centrifuged for 10 min at 4°C, and diluted 5 times with the supernatant. During sample derivatization, 40 mL of 200 mM 3-NPH (50% aqueous acetonitrile configuration, v/v) and 40 mL of 120 mM EDC-6% pyridine (50% aqueous acetonitrile configuration, v/v) were added to the feed glass vial containing the extract and refluxed for 30 min at 40°C. An injection vial with a brown injection cap is stored at −80°C until analysis, after the supernatant has been cooled on ice for 1 min and aspirated with a syringe. It is then filtered through a 0.22 m organic phase pinhole filter and stored at −80°C. Derivatization of standards: 80 μL of the standard was added to a glass feed vial, 40 μL of 200 mM 3-NPH (50 % acetonitrile aqueous configuration, v/v) and 40 μL of 120 mM EDC-6% pyridine (50 % acetonitrile aqueous configuration, v/v) were added, and the reaction was carried out at 40°C for 30 min. Cecal samples were detected qualitatively and quantitatively using UPLC-ESI-MS/MS, the same method used for sample derivatization. With the aid of triple quadrupole mass spectrometry, we quantified short-chain fatty acids in cecal samples using multireaction detection (MRM). The SCIEX OS-MQ software (Sciex, USA) identified and integrated each MRM transition automatically.

### 2.12 Proteomics of hippocampus and olfactory bulbs

#### 2.12.1 Protein extraction and digestion

Olfactory bulb (*n* = 6) and hippocampal (*n* = 8) samples from the two groups were transported on dry ice to Shanghai Jingjie Biological Company for proteomic analysis. According to the manufacturer's instructions, the following experimental procedure was used. After grinding the sample with liquid nitrogen, it was transferred to a centrifuge tube with a capacity of 5 mL. After adding four volumes of lysis buffer (8 M urea, 1% protease inhibitor cocktail) to the cell powder, a high-intensity ultrasonic processor (Scientz) was used to sonicate three times on ice. A centrifuge at 12,000 g for 10 min at 4°C removed the remaining debris. After collecting the supernatant, the protein concentration was determined using a BCA kit as directed by the manufacturer. For trypsin digestion, the protein solution was reduced with 5 mM dithiothreitol for 30 min at 56°C and alkylated with 11 mM iodoacetamide for 15 min at room temperature. For the first digestion overnight, 100 mM TEAB was added to a urea concentration less than 2 M. Trypsin was then added at a ratio of 1:50 trypsin-protein mass and then 1:00 trypsin-protein mass for a second digestion over 4 h. TMT labeling: Tryptic peptides were first dissolved in 0.5 M TEAB. Each channel of the peptide was labeled with its respective TMT reagent (based on the manufacturer's protocol, Thermo Fisher Scientific) and incubated for 2 h at room temperature. To determine labeling efficiency, five microliters of each sample were pooled, desalted, and analyzed by MS. Following the efficiency check, 5% hydroxylamine was added to quench the samples. A Strata X C18 SPE column (Phenomenex) was then used to desalt the pooled samples and a vacuum centrifuge was used to dry them. Fractionation by HPLC: Samples were fractionated by reversed-phase HPLC using an Agilent 300 Extend C18 column (5 μm particles, 4.6 mm ID, 250 mm length). As a summary, the peptides were separated in 80 fractions using a gradient of 2%- 60% acetonitrile at 10 mM ammonium bicarbonate pH 10 for 80 min. Following that, the fractions were combined into 9 fractions and then vacuum centrifuged to dry.

#### 2.12.2 LC–MS/MS analysis

The Vanquish neo ultrahigh-performance liquid phase system was used to dissolve the peptides in liquid chromatography mobile phase A and separate them. In phase A, the solution consisted of 0.1% formic acid and 2% acetonitrile in water, whereas mobile phase B was composed of 0.1% formic acid and 90% acetonitrile in an aqueous medium. The liquid phase gradient conditions were as follows: 4 min, 7%−11% B; 453 min, 11%−32% B; 5,357 min, 32%−80% B; and 5,760 min, 80% B. The peptides were separated using an ultrahigh-performance liquid phase system at a flow rate of 500 nL/min, ionized by an NSI ion source, and analyzed with an Orbitrap ExplorisTM 480 mass spectrometer from Thermo Fisher Scientific. The ion source voltage was set at 2.3 kV, the FAIMS compensation voltage (CV) was set at −45 V, and the parent peptide ions and their secondary fragments were detected and analyzed using a high-resolution Orbitrap. Primary mass spectrometer scanning range was 400–1,200 m/z, and scanning resolution was 60,000. A 110 m/z scanning range, 30,000 secondary scanning resolution, and TMTpro Reagent TurboTMT were set for the secondary mass spectrometer. The data acquisition mode utilized a data-dependent acquisition (DDA) approach, where the parent ions of the top 15 peptide segments with the highest signal intensity were sequentially chosen to enter the HCD collision pool following the initial scan. The fragmentation energy set for this process was 35%. To enhance the effective use of the mass spectrum, the automatic gain control (AGC) was configured to 100%, the signal threshold was established at 100,000 ions/s, the maximum injection time was set to Auto, and the dynamic exclusion time for consecutive mass spectrum scans was set to 30 s to prevent repeated scanning of parent ions.

#### 2.12.3 Bioinformatics analysis

The identified proteins were annotated by referring to the chromosome-level genome of C57BL/6 mice. Quantitative outcomes were utilized to compute the fold change (FC) and test the significance of differences between the two groups. Our research identified differentially expressed proteins (DEPs) with fold changes (FCs) exceeding 1.3 or < 1/1.3. The DEPs between the two groups were annotated through the Gene Ontology database, covering domains like cellular components, molecular function, and biological processes (http://geneontology.org). Additionally, we employed the eukaryotic cluster of orthologous groups (KOG) database to compare the classifications of DEPs between the two groups. The enrichment of differentially expressed proteins between the two groups was analyzed using Fisher's exact tests for GO, KEGG, protein domain, Reactome, and WikiPathways functions.

### 2.13 Data analyses

Data analysis was performed using GraphPad (8.0) and Statistical Package for the Social Sciences (SPSS 22.0). Changes in body weight and food intake were analyzed via repeated-measures ANOVA in SPSS, followed by Bonferroni's *post hoc* testing. Mouse performance in the Barnes maze and open field test was assessed using independent-sample *t*-tests to identify significant differences. Independent-sample *t*-tests were also utilized to evaluate differences in serum metabolites, cecal short-chain fatty acid concentrations, and olfactory epithelial cell counts. Normality was assessed using the Shapiro-Wilk test. For data that did not conform to a normal distribution, the non-parametric Mann-Whitney U test was employed as an alternative. Spearman correlation analysis was conducted to determine whether changes in cognition-related proteins in the hippocampus were associated with behavioral changes in the Barnes maze, as well as to identify which gut microbiota were responsible for the production of serum metabolites and short-chain fatty acids in the cecum.

## 3 Results

### 3.1 Impact of olfactory loss on body weight, food intake, and metabolism of mice

**Figure 1:**
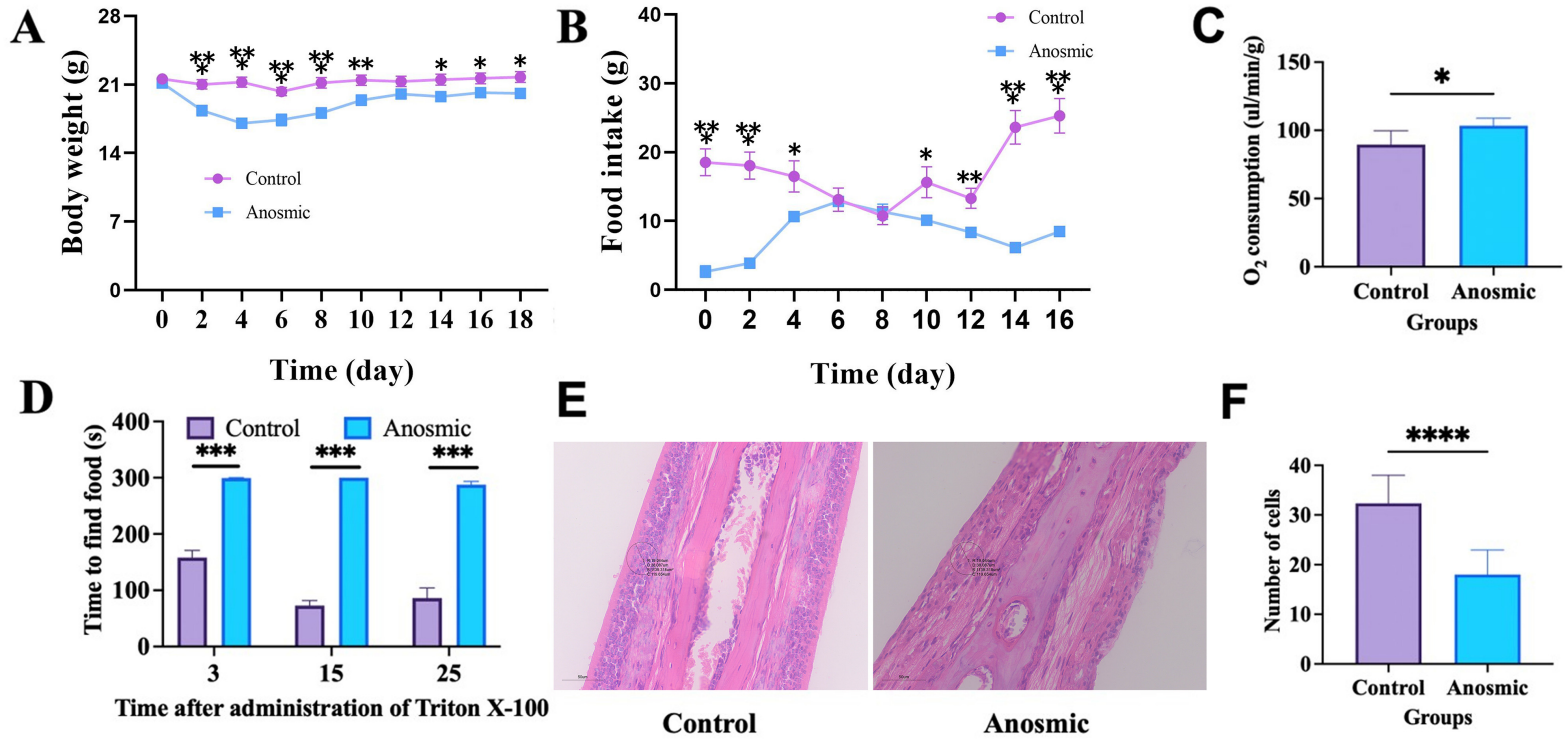
Olfactory loss and its impact on body weight, food intake, and oxygen consumption in mice. Effect of olfactory loss on body weight (line graph: gradual change along measurement) **(A)**, food intake (line graph: gradual change along measurement) **(B)**, and oxygen consumption **(C)** in mice. Time to find the food pellets after olfaction loss **(D)**. Representative image of nasal olfactory epithelial HE staining. The black circle represents the area where the cells were counted **(E)**. Changes in the nasal olfactory epithelial cell numbers **(F)**. *, **, ***, and **** represent *P* < 0.05, *P* < 0.01, *P* < 0.001, and *P* < 0.0001, respectively.

The Triton X-100 treatment had a significant effect on body weight (*F*_1, 38_ = 10.34, *P* = 0.003) and food intake (*F*_1, 38_ = 33.55, *P* < 0.001) compared to the control group (Figures 1A, B). In addition, both body weight and food intake exhibited significant changes over the course of the treatment duration (*F*_9, 342_ = 65.51, *P* < 0.001; *F*_8, 304_ = 7.60, *P* < 0.001; Figures 1A, B). Our results showed a significantly higher rate of O_2_ consumption by the anosmic group (*t* = 2.979, *df* = 10, *P* = 0.014; Figure 1C). The buried food-seeking test indicated that the mice in the anosmic group spent significantly more time to find the buried food pellets than did those in the control group 3 days (*t* = 11.26, *df* = 38, *P* < 0.0001), 15 days (*t* = 24.98, *df* = 38, *P* < 0.0001), and 25 days (*t* = 10.72, *df* = 38, *P* < 0.0001; Figure 1D). HE staining revealed a significant decrease in the number of cells in the olfactory epithelium of mice in the anosmic group compared to the control counterparts (*t* = 8.075, *df* = 34, *P* < 0.0001; Figures 1E, F).

### 3.2 Impact of olfactory loss on anxiety and cognition of mice

**Figure 2:**
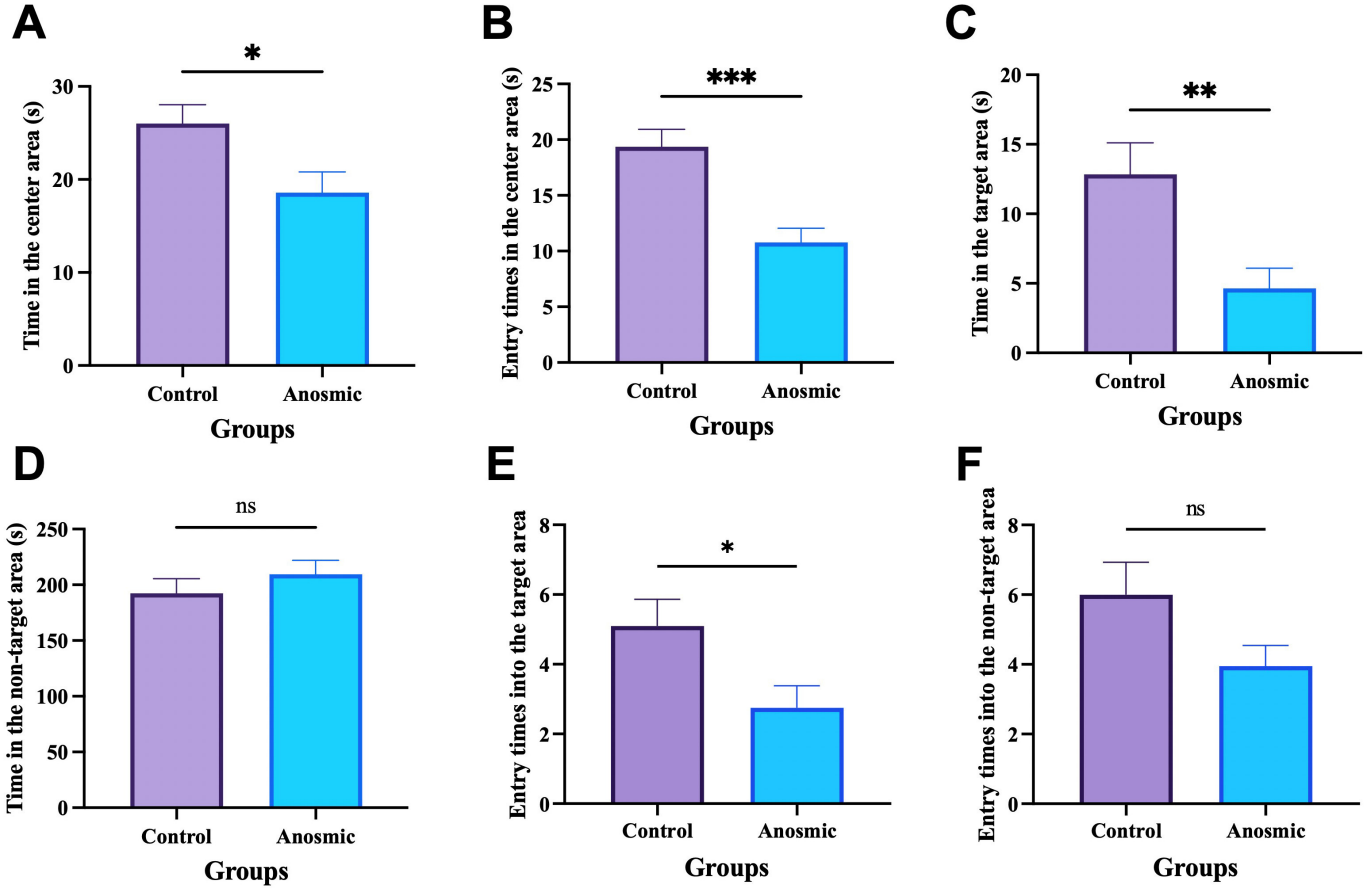
Effects of olfactory loss on anxiety behavior and cognition. Time spent in the center area of the open field-arena **(A)**. Numbers of times traversed the center of the open-field arena **(B)**. Time spent in the target zone **(C)**. Time spent in the wrong zone **(D)**. Entry times into the target area **(E)**. Entry times into the non-target area **(F)**. *, **, and *** represent *P* < 0.05, *P* < 0.01, and *P* < 0.001, respectively.

The results of the open field test showed that anosmic mice showed symptoms of anxiety-like behavior, as indicated by less time spent in the central area (*t* = 2.465, *df* = 36, *P* = 0.0186) and fewer times taken to cross the central area (*t* = 4.275, *df* = 36, *P* = 0.0001) than did the control mice (Figures 2A, B). In the Barnes maze test, the anosmic mice spent significantly less time in the target area than the control counterparts (*t* = 3.058, *df* = 38, *P* = 0.004). Moreover, time spent in the incorrect area was not different between the two groups (*t* = 0.9377, *df* = 38, *P* = 0.3543; Figures 2C, D). Moreover, mice in the C-FMT group were more likely to enter the target area than those in the A-FMT group (*t* = 2.377, *df* = 38, *P* = 0.0226; Figure 2E), while entry times into the non-target area were marginally different between the two groups (*t* = 1.861, *df* = 38, *P* = 0.0704; Figure 2F).

### 3.3 The impact of olfactory loss on the gut microbiota of mice

**Figure 3:**
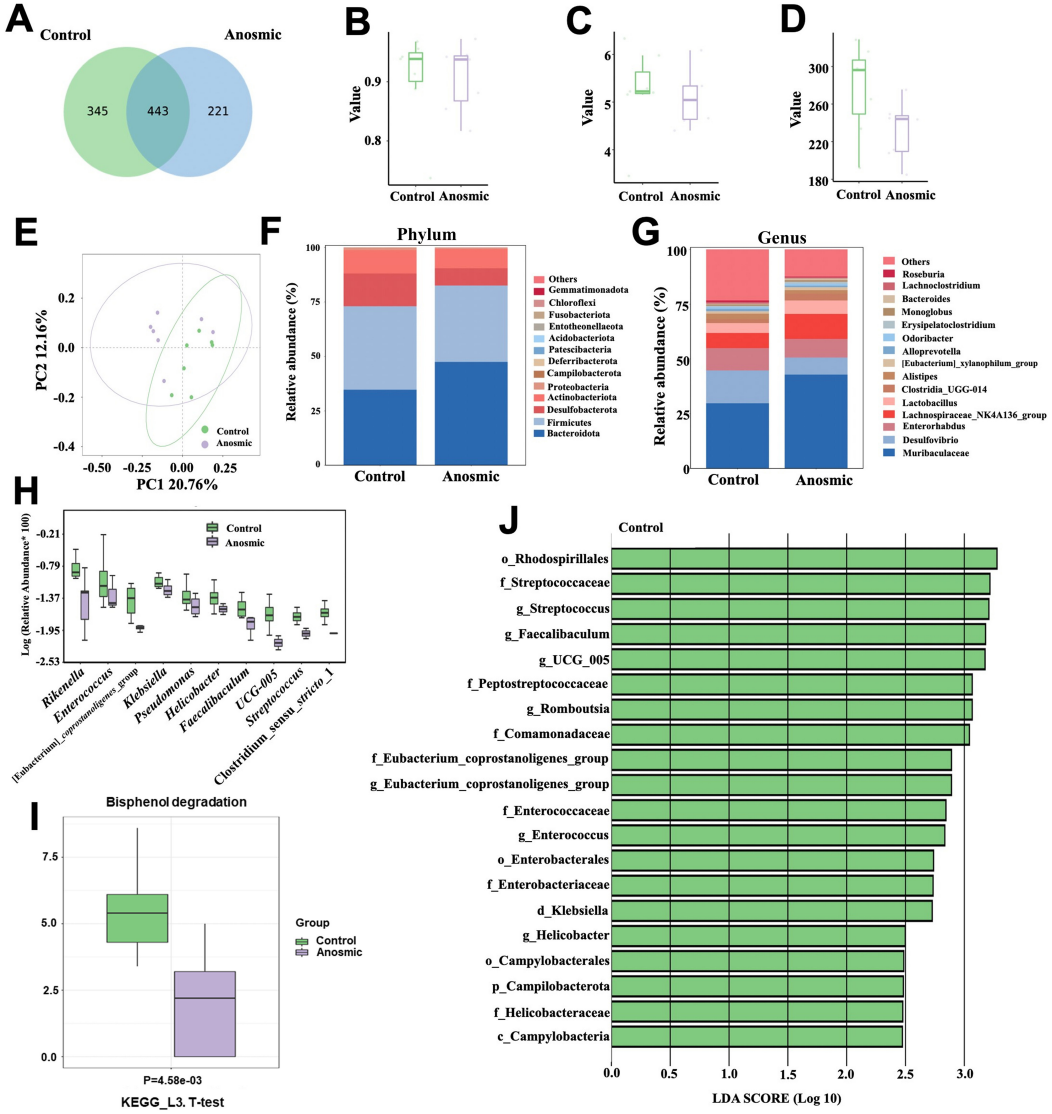
Effects of olfactory loss on the gut microbiota of mice. Venn diagram of ASV changes in the control and anosmic groups **(A)**. Simpson **(B)**, Shannon **(C)**, and Chao1 indexes **(D)** of the control and anosmic groups. PCoA of the unweighted UniFrac distance of the gut microbiota between the control and anosmic groups **(E)**. Relative abundance of the gut microbiota at the phylum **(F)** and genus **(G)** level in the control and anosmic groups. Box plot comparing the top 10 genera that changed between the control and anosmic groups **(H)**. Bisphenol degradation pathway in the KEGG pathway at level three with *P* < 0.05 **(I)**. Linear discriminant analysis (LDA) effect size (LEfSe) analysis between the control and anosmic groups **(J)**. The LDA score at the log10 scale is indicated at the bottom. The greater the LDA score is, the more significant the microbial biomarker is in the comparison.

All fecal samples reached the saturation phase, as indicated by rarefaction curves for PD and ACE, rank abundance analysis curves, and species accumulation curves (Supplementary Figures S1A–D). After removing chimeric origins, 1,009 ASVs were identified, with 221 unique to the anosmic group and 345 specific to the control group (Figure 3A). The anosmic group and the control group did not show significant differences in their Simpson, Shannon, and Chao1 indices (Figures 3B–D). Moreover, the principal coordinate analysis of diversity (PCoA) of the unweighted UniFrac distance (Anosim = 0.28, *P* = 0.025; Figure 3E) and the Bray-Curtis distance (Anosim = 0.156, *P* = 0.032; Supplementary Figure S2A) demonstrated a significant difference between the two groups. Analysis of the structural composition of the intestinal microbiota at the phylum level revealed that the dominant phyla in the anosmic and control groups were Bacteroides (47.50% vs. 34.73%, *P* = 0.27), Firmicutes (35.03% vs. 38.31%, *P* = 0.76), Desulfobacterota (7.92% vs. 15.01%, *P* = 0.06) and Actinobacteria (8.81% vs. 10.76%, *P* = 0.11) (Figure 3F). It was found that *Muribaculaceae* (42.87% vs. 29.78%, *P* = 0.21), *Desulfovibrio* (7.78% vs. 14.97%, *P* = 0.24), *Enterorhabdus* (8.47% vs. 10.2%, *P* = 0.33), and *Lachnospiraceae_NK4A136_group* (11.43% vs. 6.89%, *P* = 0.31) were the dominant genera (Figure 3G). Compared to the control group, the relative abundances of *Rikenella* (*P* = 0.047), *Enterococcus* (*P* = 0.011), *[Eubacterium]_coprostanoligenes_group* (*P* = 0.003), *Klebsiella* (*P* = 0.034), *Pseudomonas* (*P* = 0.049), *Helicobacter* (*P* = 0.012), *Faecalibaculum* (*P* = 0.014), *UCG-005* (*P* = 0.003), *Streptococcus* (*P* = 0.006), and *Clostridium_sensu_stricto_1* (*P* = 0.049) decreased significantly in the anosmic group (Figure 3H). Analysis of KEGG pathway enrichment showed significant enrichment of the bisphenol degradation signaling pathway in the control group (Figure 3I). The LEfSe analysis revealed that *Rhodospirillales, Streptococcaceae, Streptococcus*, and *Faecalibaculum* were more abundant in the control group compared to the anosmic group (Figure 3J).

### 3.4 Impact of olfactory loss on serum metabolites and short-chain fatty acids of mice

**Figure 4:**
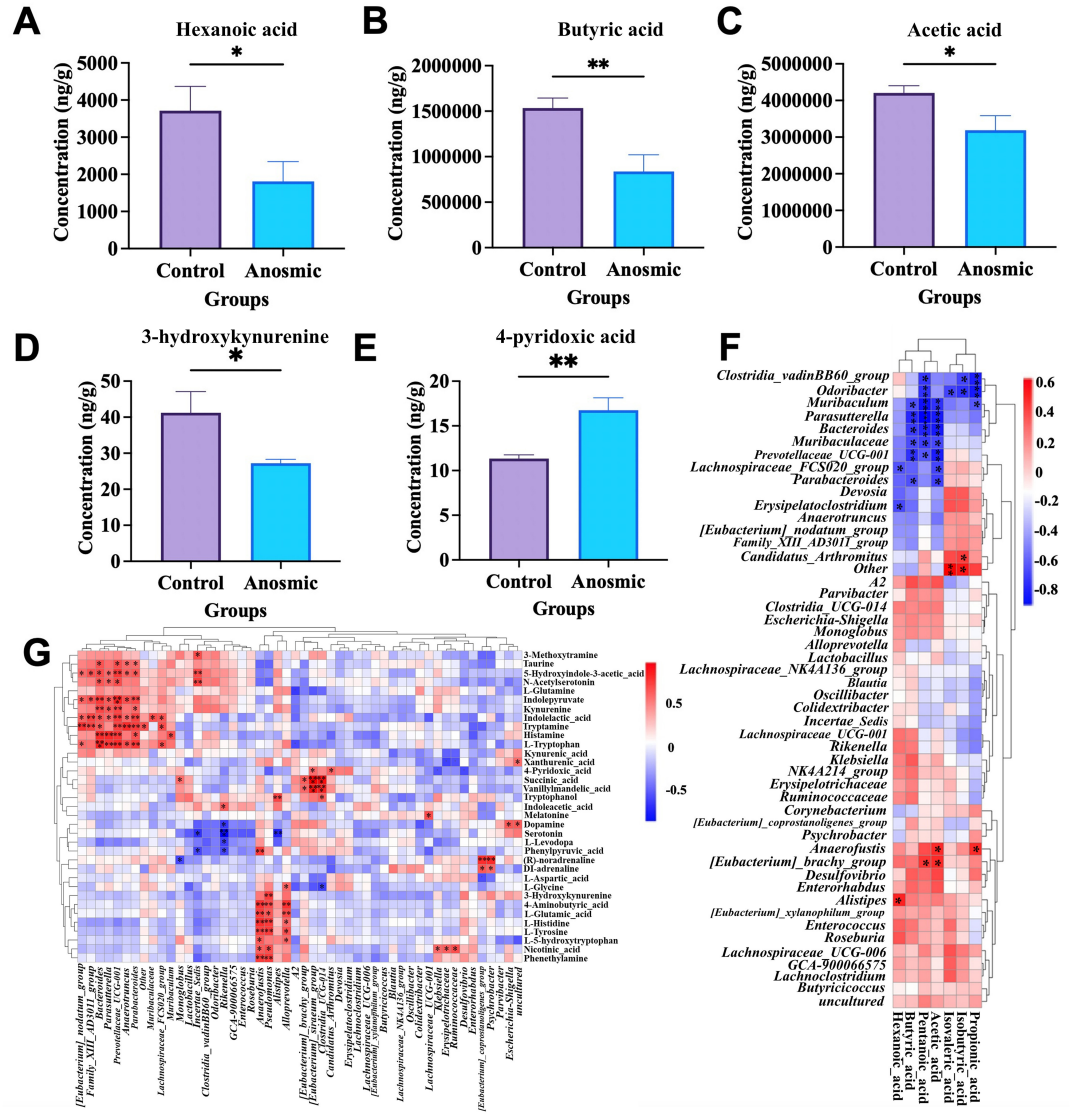
Effects of olfactory loss on cecal short-chain fatty acids and serum neurotransmitters. Effect of olfaction loss on the contents of hexanoic acid **(A)**, butyric acid **(B)**, and acetic acid **(C)**. Effect of olfaction loss on the contents of 3-hydroxykynurenine **(D)** and 4-pyridoxic acid **(E)**. Heatmap of short-chain fatty acids in conjunction with the gut microbiota **(F)**. Heatmap of the correlation between serum neurotransmitter levels and the gut microbiota **(G)**. * and ** represent *P* < 0.05 and *P* < 0.01, respectively.

The results of the short-chain fatty acid analysis indicated a significant decrease in the levels of hexanoic acid (*t* = 2.255, *df* = 12, *P* = 0.044), butyric acid (*t* = 3.248, *df* = 12, *P* = 0.007), and acetic acid (*t* = 2.298, *df* = 12, *P* = 0.04) in the anosmic group (Figures 4A–C). The analysis of the serum metabolome showed a significantly increased level of 4-pyridoxic acid (*t* = 3.801, *df* = 10, *P* = 0.004) and a decreased level of 3-hydroxykynurenine (*t* = 2.346, *df* = 10, *P* = 0.041) in the anosmic group (Figures 4D, E). The levels of butyric acid and acetic acid were negatively correlated with the abundance of *Muribaculaceae*, which was much higher in the anosmic group compared to the control group. Moreover, the level of acetic acid was positively correlated to the abundance of *Eubacterium*, which however decreased significantly in the anosmic mic (Figure 4F). We also showed that 4-pyridoxic acid was negatively correlated with the abundance of *Klebsiella* and *Desulfovibrio*, which were significantly lower in the anosmic mice. In addition, the serum level of 3-hydroxykynurenine was positively correlated with the abundance of *Pseudomonas*, which decreased significantly in the anosmic mice (Figure 4G).

### 3.5 The impact of olfaction loss on the olfactory bulb proteome

A total of 59,551 peptides were detected in the mouse hippocampus, and 57,378 peptides representing 6,565 quantifiable proteins were identified with at least one unique peptide and an FDR confidence interval of 0.01 or lower (Supplementary Figure S3A). In total, 5,715 identified proteins had molecular weights ranging from 5 kDa to 100 kDa, while 1,402 proteins exceeded 100 kDa (Supplementary Figure S3B). In addition, 676 proteins were found to have more than 20 peptides, whereas the remaining 6,441 proteins contained < 20 peptides (Supplementary Figure S3C). Moreover, the proteins identified exhibited relatively low coverage of the proteome sequence (Supplementary Figure S3D), with 2,953 proteins having over 20% sequence coverage. Furthermore, a significant portion of the proteins identified exhibited charges ranging from 2 to 3 and lengths between 7 and 20 amino acids (Supplementary Figure S3E), consistent with the influence of trypsin digestion. The Pearson correlation coefficient between samples further verified the reliability and feasibility of trypsin digestion and LC-MS/MS detection (Supplementary Figure S3F), meeting the required qualifications and demonstrating the suitability of the approach for subsequent bioinformatics analysis.

**Figure 5:**
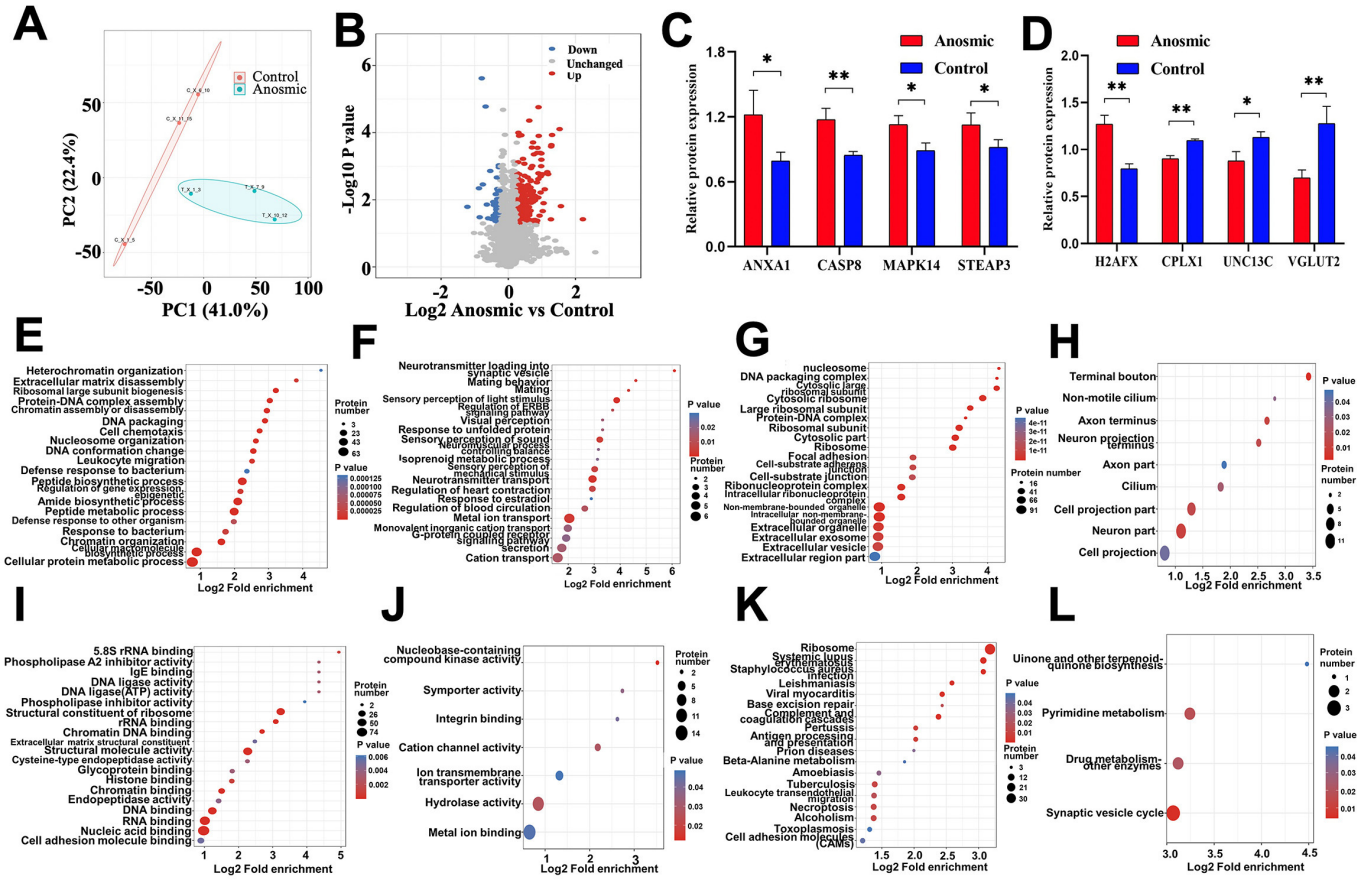
Effects of olfactory loss on the olfactory bulb proteome. PCA plot of the olfactory bulb proteome in the control group vs. the anosmic group **(A)**. Volcano plot of differentially expressed proteins between the control and anosmic groups **(B)**. Relative protein expression levels of ANXA1, CASP8, CPLX1, MAPK14, STEAP3, UNC13C, VGLUT2 and H2AFX **(C, D)**. Bubble diagram of the GO enrichment of BP-UP **(E)**, BP-DOWN **(F)**, CC-UP **(G)**, CC-DOWN **(H)**, MF-UP **(I)**, and MF-DOWN **(J)** in the control and experimental groups. The size of the blue dots indicates the number of differentially expressed proteins in the GO function, with larger dots indicating more differentially expressed proteins. Bubble diagram of the KEGG-UP **(K)** and KEGG-DOWN **(L)** enrichment in the control and anosmic groups. The size of the blue dots indicates the number of differential proteins in the KEGG pathway, and the larger dots indicate more differential proteins. * and ** represent *P* < 0.05 and *P* < 0.01, respectively.

According to the PCA results, samples from the anosmic group were significantly different from the control group (Figure 5A). The screening with an FC >1.3 criterion identified 271 proteins with differential expression between the groups; 229 proteins were notably upregulated, and 42 were notably downregulated in the anosmic group (Figure 5B). In the anosmic group, the expression levels of apoptosis-related proteins—Annexin 1 (ANXA1), Mitogen-activated Protein Kinase 14 (MAPK14), Caspase-8 (CASP8), H2A Histone Family Member X (H2AFX), and Six-Transmembrane Epithelial Antigen of the Prostate 3 (STEAP3)—were significantly upregulated (*P* = 0.0289; *P* = 0.0188; *P* = 0.004; *P* = 0.0011; *P* = 0.049), while neurotransmitter release-associated proteins Complexin1 (CPLX1), Uncoordinated-13 C (UNC13C), and Second vesicular glutamate transporter (VGLUT2) was significantly decreased (*P* = 0.0011; *P* = 0.025; *P* = 0.0045; Figures 5C, D). The results of GO functional enrichment analysis revealed that 20 functions were significantly upregulated and another 20 functions were significantly downregulated (Figures 5E, F) in BP; 20 functions were significantly upregulated and 9 functions were significantly downregulated in CC (Figures 5G, H); and 20 functions were significantly upregulated and 7 functions were significantly downregulated in MF (Figures 5I, J). KEGG pathway enrichment analysis revealed that 18 pathways were significantly upregulated and 4 pathways were significantly downregulated (Figures 5K, L), including necroptosis signaling pathway (Supplementary Figure S5A) and synaptic vesicle cycle signaling pathway (Supplementary Figure S5B).

### 3.6 Impact of olfactory loss on the hippocampal proteome

There were 35,997 peptides detected in the hippocampal region of the mice, and 34,309 peptides covering 4,259 quantifiable proteins were further identified with at least one unique peptide and an FDR confidence interval of 0.1 (Supplementary Figure S4A). The molecular weights of 4,098 identified proteins ranged from 5 kDa to 100 kDa, while 935 proteins exceeded 100 kDa (Supplementary Figure S4B). Among the remaining 4,719 proteins, 314 contained more than 20 peptides, and 314 contained fewer than 20 peptides (Supplementary Figure S4C). The proteome sequence coverage of the identified proteins was relatively low (Supplementary Figure S4D), with 1,805 proteins covering over 20% of the proteome. Furthermore, most detected proteins ranged between 2–3 charges and 7 to 20 amino acids in length (Supplementary Figure S4E), which is consistent with trypsin digestion. By using Pearson correlation coefficients between samples, we can further verify the reliability and feasibility of digestion with trypsin and detection with LC-MS/MS (Supplementary Figure S4F), meeting the required qualifications and demonstrating the suitability of the approach for subsequent bioinformatics analysis.

**Figure 6:**
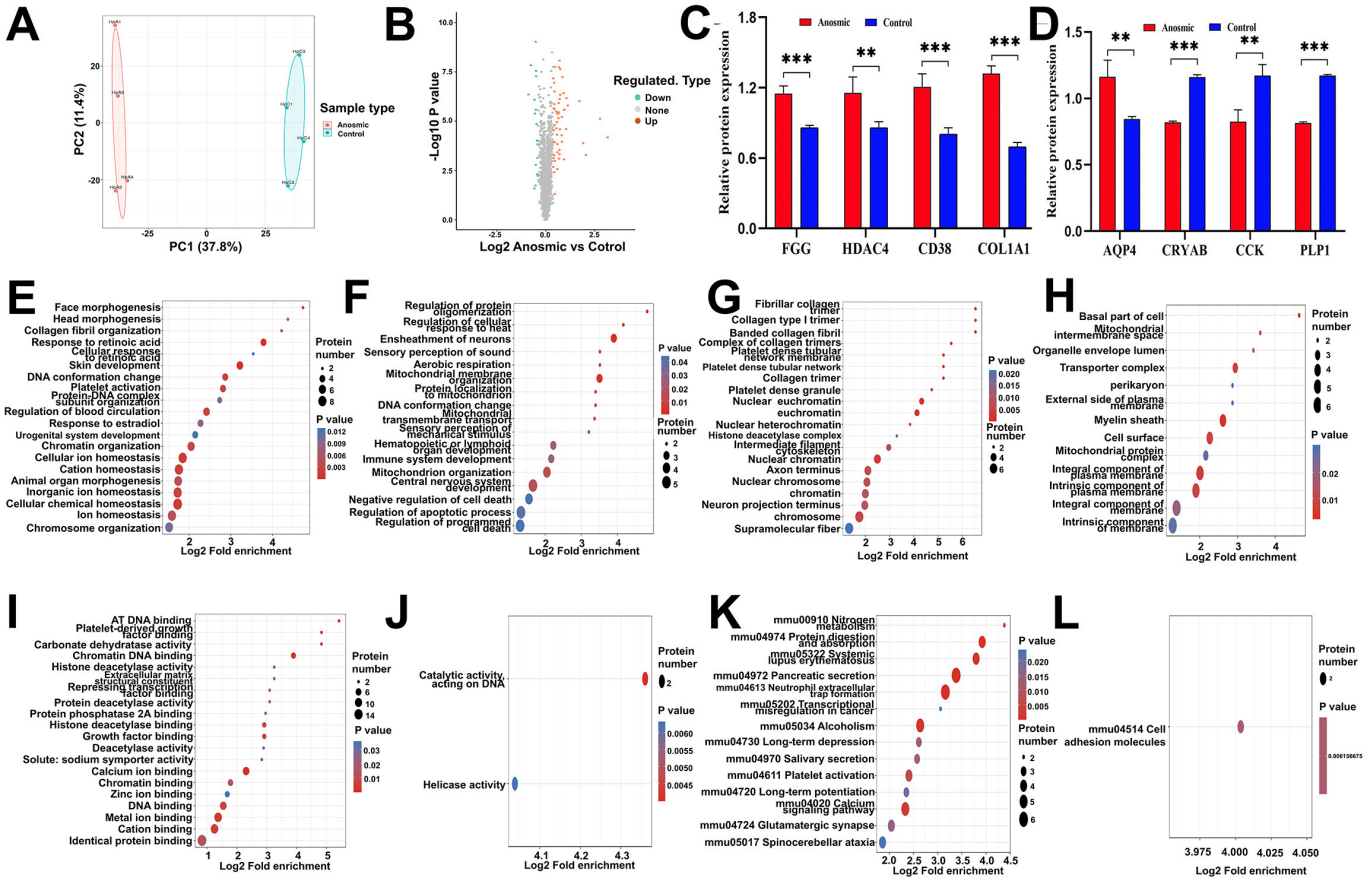
Effects of olfactory loss on the hippocampal proteome. PCA plot of the hippocampal proteome in the control group vs. the anosmic group **(A)**. Volcano plot of differentially expressed proteins between the control and anosmic groups **(B)**. Relative expression levels of AD-associated proteins FGG, HDAC4, CD38, COL1A1, AQP4, CRYAB, CCK and PLP1 **(C, D)**. Bubble diagram of the GO enrichment of BP-UP **(E)**, BP-DOWN **(F)**, CC-UP **(G)**, CC-DOWN **(H)**, MF-UP **(I)**, and MF-UP **(J)** in the control and anosmic groups. The size of the blue dots indicates the number of differentially expressed proteins in the GO function, with larger dots indicating more differentially expressed proteins. Bubble diagram of the KEGG-UP **(K)** and KEGG-DOWN **(L)** enrichment in the control and anosmic groups. The size of the blue dots indicates the number of differential proteins in the KEGG pathway, and the larger dots indicate more differential proteins. *, **, and *** represent *P* < 0.05, *P* < 0.01, and *P* < 0.001, respectively.

According to the PCA results, samples from the anosmic group were significantly different from the control group (Figure 6A). In total, 75 proteins were differentially expressed between the two groups based on FC >1.3; 56 proteins were significantly upregulated, and 19 proteins were significantly downregulated in the anosmic group (Figure 6B). In the anosmic group, the expression of Procollagen, type I, α1 (COL1A1 up-regulated in cognitive impairment), Aquaporin-4 (AQP4 plays a role in the proinflammatory features of astrocytes), Fibrinogen gamma chain (FGG regulate nervous system functions), ADP-Ribosyl Cyclase 1(CD38 cognition and spatial memory related), and Histone deacetylase 4 (HDAC4 cognition and spatial memory related) was significantly increased (*P* < 0.0001; *P* = 0.001; *P* = 0.0005; *P* = 0.0003; *P* = 0.0038). Conversely, the expression levels of proteins related to neurological function—Alpha-Crystallin B Chain (CRYAB), Cholecystokinin (CCK; acting as a neurotransmitter and growth factor), and Myelin Proteolipid Protein (PLP1; essential for myelin compaction and interperiod dense line formation)-were significantly downregulated (*P* < 0.0001; *P* = 0.0016; *P* < 0.0001; Figures 6C, D). The results of GO functional enrichment analysis revealed that 20 functions were significantly upregulated and 17 functions were significantly downregulated in BP (Figures 6E, F); 20 functions were significantly upregulated and 13 functions were significantly downregulated in CC (Figures 6G, H); and 20 functions were significantly upregulated and 2 functions were significantly downregulated in MF (Figures 6I, J). KEGG pathway enrichment analysis showed that 14 pathways were significantly upregulated, but 1 pathway significantly downregulated (Figures 6K, L). The differentially expressed proteins were primarily associated with calcium signaling pathways, long-term depression, neutrophil extracellular trap formation, and spinocerebellar ataxia signaling pathways (see Supplementary Figures S6A–D).

### 3.7 Impact of FMT on the gut microbiota of mice

**Figure 7:**
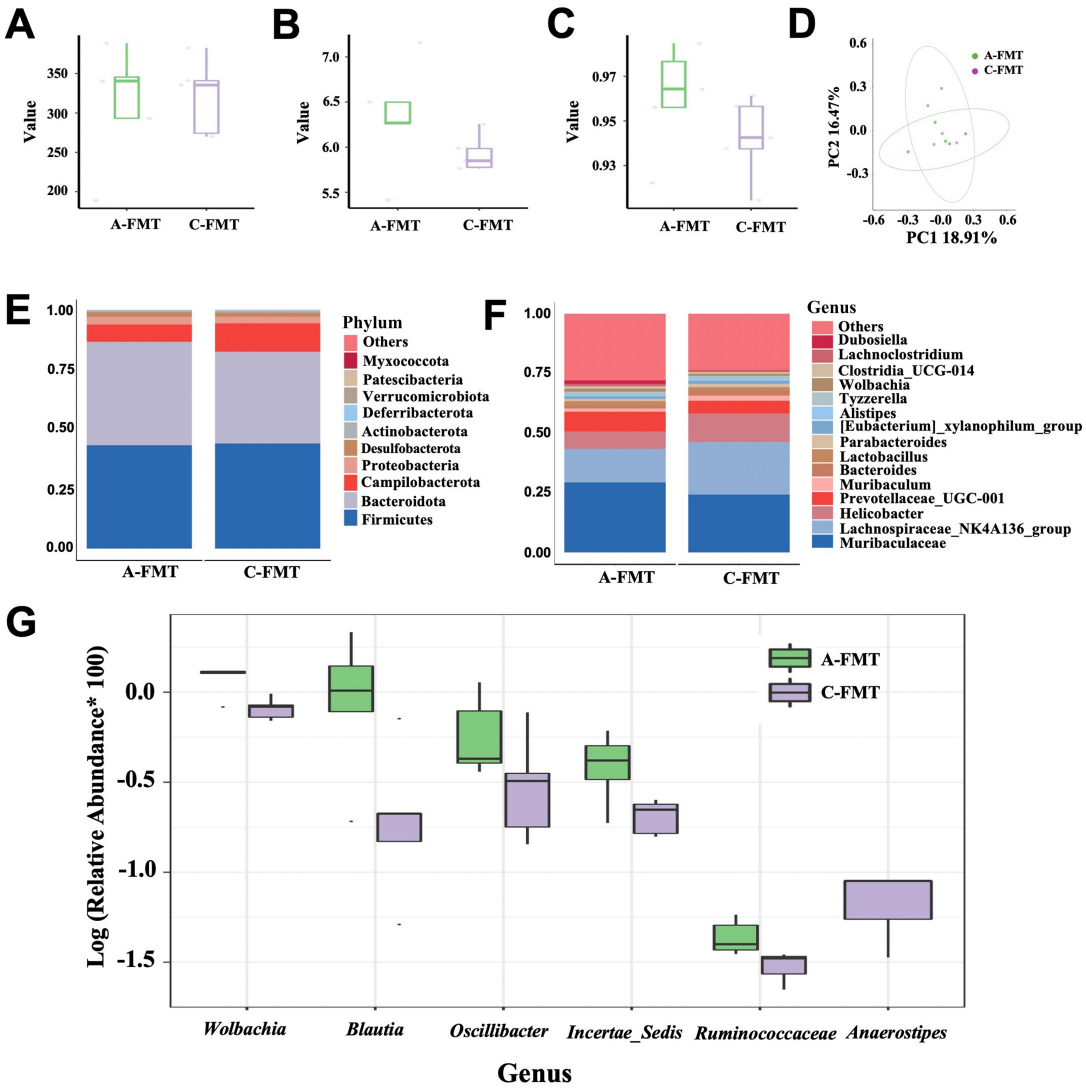
Effect of FMT on the gut microbiota of C57BL/6 mice. Chao1 **(A)**, Shannon **(B)**, and Simpson **(C)** indexes of the gut microbiota of the C-FMT and A-FMT groups. PCoA of the unweighted UniFrac distance of the gut microbiota between the C-FMT and A-FMT groups **(D)**. Relative abundance of the gut microbiota at the phylum **(E)** and genus **(F)** levels in the C-FMT and A-FMT groups. Box plot comparing the top 10 genera that changed significantly between the C-FMT and A-FMT groups **(G)**.

There were no significant differences in the Chao1, Shannon, and Simpson indices between A-FMT and C-FMT groups (Figures 7A–C). In addition, PCoA of the unweighted UniFrac distance (Anosim = 0.028, *P* = 0.37; Figure 7D) and the Bray-Curtis distance (Anosim = 0.052, *P* = 0.33; Supplementary Figure S2B) showed no significant differences between the A-FMT and C-FMT groups. Microbiota composition analysis revealed that the dominant phyla of the A-FMT and C-FMT groups were Firmicutes (43.30% vs. 44.01%, *P* = 0.58), Bacteroidota (43.30 vs. 38.45%, *P* = 0.45), Campilobacterota (7.28% vs. 11.92%, *P* = 0.34) and Proteobacteria (3.21% vs. 2.72%, *P* = 0.31) (Figure 7E). The dominant genera in the A-FMT and C-FMT groups were *Muribaculaceae* (29.36% vs. 24.26%, *P* = 0.25), *Lachnospiraceae_NK4A136_group* (14.07% vs. 22.05%, *P* = 0.60), *Helicobacter* (7.27% vs. 11.92%, *P* = 0.33), and *Prevotellaceae_UCG-001* (8.26% vs. 5.34%, *P* = 0.32) (Figure 7F). At the genus level, the relative abundances of *Ruminococcaceae* (*P* = 0.001), *Wolbachia* (*P* = 0.003), *Klebsiella* (*P* = 0.028), *Incertae_Sedis* (*P* = 0.037), *Blautia* (*P* = 0.038), and *Oscillibacter* (*P* = 0.042) increased significantly in the A-FMT group, while the relative abundance of *Anaerostipes* (*P* = 0.023) decreased significantly in the A-FMT group (Figure 7G).

### 3.8 Impact of FMT on the cognitive ability of mice

**Figure 8:**
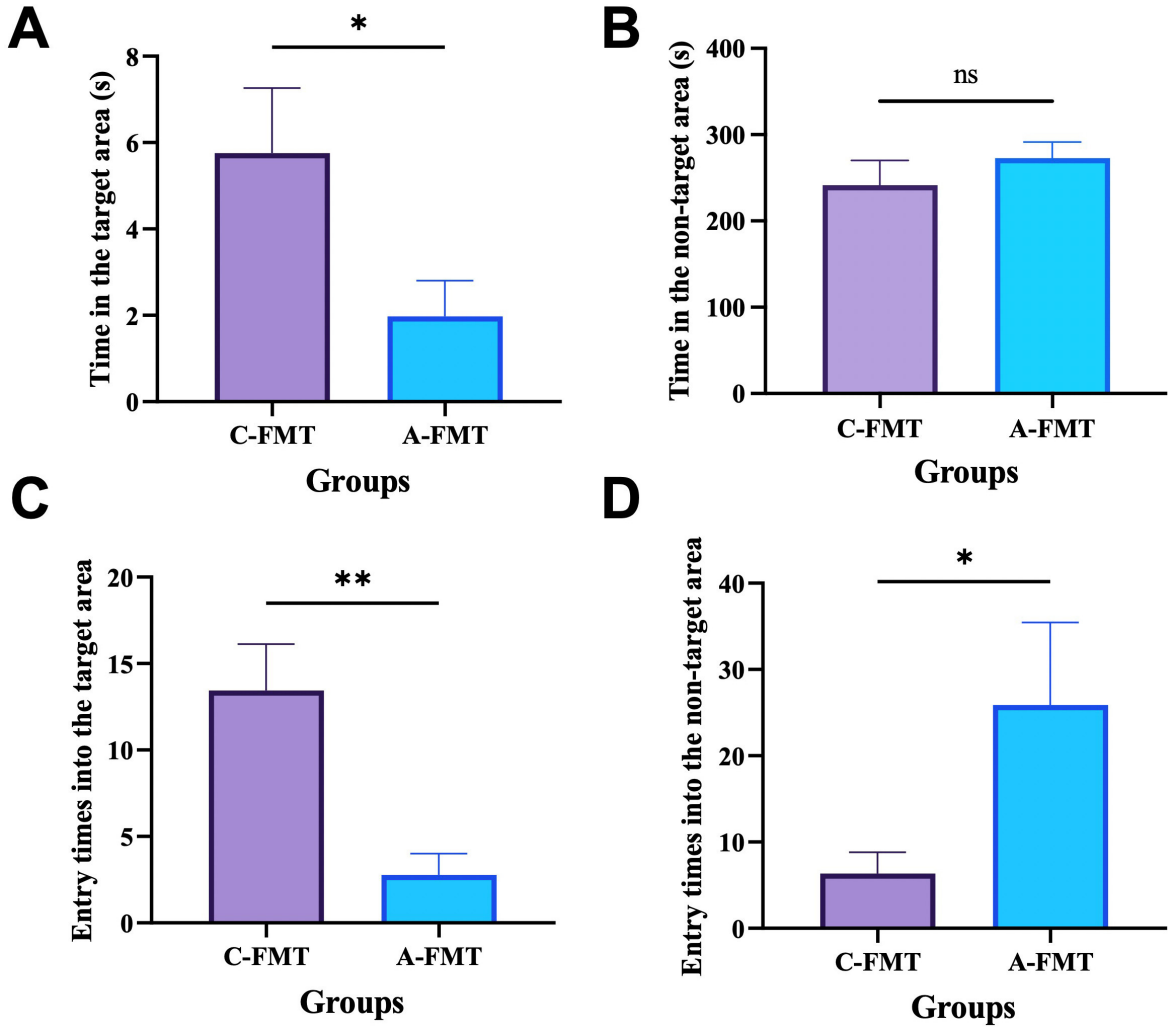
Effect of FMT on the cognitive ability of mice. Effect of FMT on the time spent by the mice in the target zone **(A)** and the wrong zone **(B)**. Entry times into the target area **(C)**. Entry times into the non-target area **(D)**. * and ** represent *P* < 0.05 and *P* < 0.01, respectively.

Mice in the C-FMT group spent much more time in the target area than those in the A-FMT group (*t* = 2.200, *df* = 16, *P* = 0.0428; Figure 8A). However, mice in the C-FMT group spent similar time in the non-target area to those in the A-FMT group (*t* = 0.912, *df* = 16, *P* = 0.3752; Figure 8B). Moreover, we found that mice in the C-FMT group were more likely to enter the target area than those in the A-FMT group (*t* = 3.630, *df* = 16, *P* = 0.0023; Figure 8C), while mice in the C-FMT group were less likely to enter the non-target area than those in the A-FMT group (*t* = 2.512, *df* = 16, *P* = 0.0249; Figure 8D).

### 3.9 Correlation analysis between hippocampal proteins and behavioral performance in the Barnes maze

The relative expression levels of proteins AQP4 (*R* = −0.5030, *P* = 0.2089), CD38 (R = −0.4311, *P* = 0.2854), HDAC4 (*R* = −0.5150, *P* = 0.1977) and COL1A1 (*R* = −0.5509, *P* = 0.1627) showed no significant correlation with the time spent in the target zone. In contrast, FGG (*R* = −0.8264, *P* = 0.0172) exhibited a negative correlation with the time spent in the target zone (Supplementary Figure S7A). Similarly, the relative protein expression levels of CCK (*R* = 0.5868, *P* = 0.1354), PLP1 (R = 0.4192, *P* = 0.3013), and CRYAB (R = 0.6145, *P* = 0.1135) did not correlate significantly with the time spent in the target zone (Supplementary Figure S7B).

## 4 Discussion

In this study, we observed preliminary associations between olfactory loss in mice and trends toward reduced body weight, decreased food intake, and elevated oxygen consumption. Additionally, our findings suggest a potential correlation between olfactory impairment and mild cognitive differences or anxiety-like behaviors. These observations might align with shifts in gut microbiota composition and fluctuations in neurotransmitter and short-chain fatty acid levels. While the mechanisms remain unclear, detected changes in cognition-related hippocampal proteins offer initial support for the possibility that interactions within the microbiota-gut-brain axis could contribute to the relationship between olfactory dysfunction and subtle cognitive or behavioral changes. Further research is needed to clarify these connections.

### 4.1 Effect of olfactory loss in mice

Olfaction, the most primeval and volatile sense (Harel et al., 2003), plays a vital role in the perception of flavor. Sense of smell is closely linked to appetite and food intake, allowing mammals to detect and discriminate thousands of different odors, including those from food. Food odor can trigger hunger and increase food intake because olfaction plays a significant role in modulating appetite and food consumption (Sharma et al., 2019). Our results clearly showed Triton X-100 successfully induced olfaction loss, as indicated by the facts of decreased efficiency of food pellet searching, attenuation of the olfactory epithelium, and alterations in the proteome of olfactory bulbs. Moreover, olfaction loss induced decrease in food uptake and loss of body weight and subsequently led to oxygen metabolism abnormality in mice, which has been identified as the main syndrome of AD (Ogawa et al., 1996; Gillette-Guyonnet et al., 2000; Saha et al., 2016). Most importantly, our findings provide evidence that olfactory loss induces cognitive impairment and anxiety-like behavior, thereby reinforcing previous studies demonstrating that olfactory dysfunction affects mood and cognition (Sarafoleanu et al., 2009) or may even lead to depression (Kim and Bae, 2022). These results corroborate our first hypothesis that olfactory dysfunction can induce cognitive deficits and anxiety-like behavior in mice.

### 4.2 Olfaction loss, gut microbiota and cognition in mice

Although the link between olfactory and cognitive function has been studied extensively, most of these studies have focused on how the improvement of olfactory function ameliorates cognitive function, possibly because of the intrinsic neural connections between the olfactory and cognitive systems (Salimi et al., 2022; Chen et al., 2023). The gut microbiota, which is the community of microorganisms living in gastrointestinal tract, has been increasingly recognized for its role in various aspects of animal health, including metabolism, immune function, and even cognitive ability. A strong link between olfaction and the gut microbiota has been well reported (Bienenstock et al., 2018), i.e., gut microbiota regulates olfaction and then appetite and energy balance. We instead showed that olfactory impairment alters gut microbiota of mice possibly due to significant decreases in appetite and food uptake. This speculation can be supported by the facts that fasting or dietary restriction has greatly altered gut microbiota of mice (Li L. et al., 2020) and rats (Teker and Ceylani, 2023). Alterations in gut microbiota have been reported to be closely linked to cognition ability (Feng et al., 2023) and mood changes, e.g., anxiety and depression (Mitrea et al., 2022). It seems unclear whether the changes in gut microbiota are a cause or a symptom of mood changes. In this study, we showed that alterations in the gut microbiota of the anosmic mice echo those observed in the AD patients. Specifically, the abundances of *Rikenella, Faecalibaculum* and *Clostridium_sensu_stricto_1*, which have been shown to be significantly depleted in the AD mice (Chen et al., 2021; Li et al., 2023), and dementia patients (Wanapaisan et al., 2023), were significantly decreased in the anosmic group. Moreover, the abundance of Desulfobacterota, which mainly includes *Desulfovibrio* that can confer resilience to anxiety-like behavior in a mouse model (Wu et al., 2023), decreased marginally in the anosmic group. Therefore, alterations of gut microbiota induced by olfaction loss through decreasing food intake may represent a pathway to regulate the cognitive ability and anxiety behavior in mice via regulation of short-chain fatty acids (Yang et al., 2022). For FMT mice, the relative abundance of *Anaerostipes* was downregulated in the A-FMT group. Anaerostipes is capable of utilizing dietary inositol for the production of short-chain fatty acids (SCFAs), which beneficially modulate both the peripheral and central nervous systems (Bui et al., 2021). Studies have shown that *Anaerostipes* is downregulated in Alzheimer's disease (AD) patients (Zhao et al., 2025). These findings are consistent with the performance of A-FMT mice in the Barnes maze test. Additionally, although not reaching a significant difference, we observed a reduction in the abundance of *Rikenella* and *Clostridium sensu stricto 1* in the A-FMT group, which aligns with the trend observed in the Anosmic group.

However, alterations in olfactory function can affect brain function in a number of ways, such as the neural connections we mentioned earlier. Study showed that odor sensations processed in the central nervous system may induce pleasant reactions, positive mood and emotions, but also negative responses (Guadalupe-Fernandez et al., 2021). Therefore, we cannot attribute the cognitive and emotional changes following olfactory loss to alterations in the gut microbiota; there may be bidirectional regulation. Not only that, but the relationship between the brain and the gut microbiota is also bidirectional, and changes in relevant functional areas of the brain can also be involved in the regulation of the gut microbiota through a variety of pathways, such as neurological-neurological, neuroendocrine and neuroimmune pathways (Wang and Wang, 2016; Rathore et al., 2025). Therefore, the causality of the changes in the gut microbiota and related behaviors following the loss of olfaction in this study is not unique, and further studies are still needed. However, it is undeniable that alterations in the gut microbiota play an integral role in the cognitive-emotion changes induced by olfactory loss. The changes in gut microbiota composition and cognitive ability of recipient mice in the Barnes maze provide support for our second hypothesis: olfactory loss-induced alterations in gut microbiota play a critical role in the observed effects.

### 4.3 Changes in short-chain fatty acids and serum neurotransmitters

Along with significant alterations of gut microbiota in mice, we observed distinct changes in SCFAs between the control and anosmic groups. As the main metabolites of gut microbiota, SCFAs have been hypothesized to serve as alternative substrates for energy metabolism to counteract the brain hypo-metabolism to ameliorate AD (Zilberter and Zilberter, 2017). Disorders of short-chain fatty acid metabolism can directly affect hippocampal function (Dalile et al., 2019; Xu et al., 2023). SCFAs also have the potential to modulate the maturation and function of microglia in the brain to alleviate AD (Colombo et al., 2021). However, the level of SCFAs has always been shown to decrease in the feces of patients with mild cognitive impairment and AD patients (Nagpal et al., 2019; Wu et al., 2021). On the contrary, supplementation of SCFAs has been observed to alleviate AD syndrome via gut microbiota-brain axis (Martins and Fernando, 2014). In our study, the contents of cecal short-chain fatty acids (e.g., butyric acid, acetic acid, and hexanoic acid), which are strongly associated with cognition (Du et al., 2022; Fan et al., 2023; Gao et al., 2023a), were significantly decreased in the anosmic mice with olfactory dysfunction. These patterns are identical to those observed in the AD patients (Wu et al., 2021; Chen et al., 2022). Moreover, the metabolism of cecal short-chain fatty acids is highly dependent on gut microbiota, as indicated by the joint analyses between 16S rDNA and targeted metabolome in our results. Therefore, our study suggests that the decreased production of gut SCFAs in the anosmic mice may represent a biological mechanism through which the gut microbiota induce cognitive impairment and anxiety behavior in mice.

In addition to short-chain fatty acids, neurotransmitters synthesized by the gut microbiota are also crucial in regulating brain function (Lyte, 2014). Previous study has confirmed that several neurotransmitter systems, including the cholinergic, somatostatinergic, serotonergic, noradrenergic, and even dopaminergic neurons, are disturbed in AD (Reinikainen et al., 1990). Moreover, an imbalance of several neurotransmitters is evident in the brains of AD patients (Snowden et al., 2019). The results of numerous studies confirm that neurotransmitters, which play an important role in the regulation of synaptic plasticity, are significantly altered in the progression and pathogenesis of AD (Yang et al., 2023). In our study, we observed significant decreases in serum 3-hydroxykynurenine but increases in 4-pyridoxic acid in the anosmic group. Previous study shows clear evidence for the antioxidant role of 3-hydroxykynurenine in the brain (Leipnitz et al., 2007). However, the level of 3-hydroxykynurenine has ever been observed to decline in AD (Giil et al., 2017). Although the precise role of 4-pyridoxic acid remains to be elucidated, its levels have been demonstrated to be elevated in patients with Alzheimer's disease (AD) (Nielsen et al., 2021). The findings from our integrated analyses further reinforce the pivotal role of gut microbiota in modulating serum neurotransmitter metabolism. Based on these observations, we propose that the imbalance of specific neurotransmitters, driven by alterations in gut microbiota composition, may significantly contribute to the cognitive impairment and anxiety-like behavior induced by olfactory dysfunction in our study. Nevertheless, given that we did not perform additional experiments on the FMT group mice, our results provide only partial support for our hypothesis, which constitutes a limitation of this study.

### 4.4 Impact of olfactory loss on brain function

The results of the olfactory bulb proteome showed that the expression levels of olfactory proteins, such as ANXA1, MAPK14, CASP8, STEAP3, and H2AFX, were significantly elevated in the anosmic mice. The elevation of these proteins has been evidenced to be closely associated with apoptosis (Li et al., 2016; Aghababazadeh et al., 2017). Increased levels of apoptosis are an important feature of cognition decline (Sharma et al., 2021), while reduced levels of apoptosis are usually correlated with improved neuronal function (Wang et al., 2019). We also found that the expression level of CPLX1, UNC13C, and VGLUT2 was reduced, indicating synaptic dysfunction (Shigeo et al., 2001; Kielar et al., 2012; Ansari et al., 2023). Thus, olfactory loss may be caused by the activation of the necroptosis and synaptic vesicle cycle signaling pathways, leading to the dysfunction of olfactory bulbs in our study.

The hippocampus plays a crucial role in memory formation and consolidation, as well as spatial navigation (Chettih et al., 2024). Among the proteins detected in the hippocampus, COL1A1, AQP4, FGG, CD38, and HDAC4 were significantly upregulated. Increased expression of COL1A1 has been observed in senescence-accelerated mice compared with controls (Alvarez-López et al., 2013), indicating a biomarker of cognitive impairment. Evidence has shown that expression of AQP4, the most abundant water channel in the central nervous system, is significantly higher in the brain of AD patients than in that of controls (Moftakhar et al., 2010), suggesting its potential role as a biomarker of cognition decline (Bergström et al., 2021). Moreover, increased expression of AQP4 has been considered as a sign of brain aging (Zeppenfeld et al., 2017). FGG, which has been recognized as the marker for inflammatory responses, plays a role in neurodegenerative diseases (Maierhaba et al., 2018). Previous studies have demonstrated that cognition impairment is attenuated in a CD38-deficient mouse model (Blacher et al., 2015). Moreover, CD38 knockout mice are protected from neurodegenerative and neuroinflammatory insults (Guerreiro et al., 2020). Aberrant expression of HDAC4, which represses genes essential for synaptic function (Sando et al., 2012), plays a pivotal role in cognitive impairment. Therefore, upregulation of those hippocampal proteins is supposed to be closely related to the cognitive impairment and anxiety behavior of mice subjected to olfaction loss. Moreover, we observed decreased CRYAB along with impaired cognitive function in mice, which has been evidenced in previous studies (Do Carmo et al., 2018; Gao et al., 2023b). CCK, which allows the uptake of fat and fatty acids (Plagman et al., 2019), predicts better cognitive outcomes at higher concentrations in the hippocampus. However, CCK decreased significantly together with PLP1 in the hippocampus in the anosmic mice. As an important protein in myelination in the hippocampus, downregulation of PLP1 has been observed in AD prefrontal cortex (Saura et al., 2023). Collectively, the downregulation of CRYAB, CCK, and PLP1 appears to play a critical role in the cognitive impairment and anxiety-like behavior observed in anosmic mice in the present study. The results of the correlation analysis indicated that the time spent in the target zone (Barnes maze) by C57 mice was significantly correlated with the relative expression of FGG protein. These findings further suggest that alterations in hippocampal proteins may represent a mechanism underlying olfactory loss-induced cognitive deficits.

Apart from alterations of hippocampal proteins, we detected several key pathways that have been upregulated in the hippocampus of the anosmic mice. Calcium signaling is involved in regulating learning and memory, and therefore derangement in calcium signaling plays a significant role in numerous neurodegenerative diseases (Egorova et al., 2015). Upregulation of the calcium signaling pathway in our study is believed to be related to an increase in the concentration of calcium ions in hippocampal tissue (Sushma and Mondal, 2019). The activation of long-term depression, also observed in our study, has been proven to be closely related to AD (Trillaud-Doppia et al., 2016; Mango et al., 2019). Neutrophils, known to release neutrophil extracellular traps, have been observed in multiple brain regions (e.g., hippocampus and olfactory bulbs) in AD models. Increased release of neutrophil extracellular traps by neutrophils has been found to exacerbate the AD-like pathology and cognitive decline (Zenaro et al., 2015; Aries and Hensley-Mcbain, 2023). Neutrophil extracellular traps are also closely associated with neuroinflammation (Shafqat et al., 2023), and therefore activation of this signaling pathway may predict increased levels of neuroinflammation in the hippocampus of the anosmic group. Spinocerebellar ataxias, representing a group of progressive hereditary neurodegenerative diseases (Watase et al., 2007; Egorova et al., 2015), was activated in the anosmic group, possibly reflecting neuronal degeneration in the hippocampus. Therefore, the activation of these signaling pathways due to olfactory loss may represent the primary molecular mechanisms underlying the cognitive impairment and anxiety-like behavior observed in anosmic mice. Our final hypothesis is that changes in hippocampal proteins and pathways, which underlie the cognitive deficits induced by olfactory loss, can be substantiated by the above findings.

### 4.5 Limitations of this study

In this study, we demonstrated that the alterations in gut microbiota and cognitive impairment may potentially contribute to olfactory loss. We concur that olfactory impairment may directly disrupt brain regions associated with emotion and cognition, given the intrinsic association between olfaction and central nervous system function. Furthermore, bidirectional communication within the brain-gut-microbiota axis via neural, endocrine, and immune pathways implies that alterations in brain function can reciprocally modulate gut microbiota composition. However, as we did not conduct additional cognitive experiments in the FMT group, we cannot definitively conclude whether cognitive dysfunction induced by olfactory loss occurs exclusively through the gut microbiota pathway. Furthermore, future studies should consider expanding the sample size of the experiment to enhance the robustness and generalizability of the findings. Nevertheless, there is no doubt that the link between olfactory dysfunction and cognition holds significant research value, particularly for the early diagnosis of neurodegenerative diseases, mechanistic investigations, and the development of intervention strategies. Future studies should dig deeper into the molecular mechanisms underlying the olfactory-cognitive connection from multiple perspectives.

### 4.6 Conclusion

In this study, we utilized a comprehensive approach involving behavioral assessments, 16S rDNA sequencing, targeted metabolomics, TMT proteomics, and fecal microbiota transplantation to explore the potential links between olfactory function and cognitive changes in mice. Our findings indicate that olfactory loss may contribute to subtle cognitive impairments and anxiety-like behaviors. Notably, preliminary results reveal associations between olfactory dysfunction and alterations in gut microbiota composition, modest upregulation of cognition-related proteins, activation of specific hippocampal pathways, and fluctuations in intestinal short-chain fatty acids and serum neurotransmitters. Collectively, these observations support the hypothesis that olfactory dysfunction may influence cognitive changes via interactions within the metabolism-gut-microbiota-brain axis. To the best of our knowledge, this study provides initial evidence for the mechanisms underlying the connection between olfactory dysfunction and cognitive/behavioral alterations, offering potential directions for further investigation in Alzheimer's disease (AD) research through the microbiota-gut-brain axis.

## Data Availability

The original contributions presented in the study are publicly available in the Figshare Dryad Digital Repository. This data can be found here: https://doi.org/10.6084/m9.figshare.26340262.

## References

[B1] Adams D. R. Kern D. W. Wroblewski K. E. Mcclintock M. K. Dale W. Pinto J. M. (2018). Olfactory dysfunction predicts subsequent dementia in older US adults. J. Am. Geriatr. Soc. 66, 140–144. 10.1111/jgs.15048 28944467 PMC6317879

[B2] Aghababazadeh M. Dorraki N. Javan F. A. Fattahi A. S. Gharib M. Pasdar A. (2017). Downregulation of Caspase 8 in a group of Iranian breast cancer patients – A pilot study. J. Egypt Natl. Canc. Inst. 29, 191–195. 10.1016/j.jnci.2017.10.001 29233452

[B3] Alvarez-López M. J. Castro-Freire M. Cosín-Tomás M. Sanchez-Roige S. Lalanza J F. Del Valle J.. (2013). Long-term exercise modulates hippocampal gene expression in senescent female mice. J. Alzheimer's Dis. 33, 1177–1190. 10.3233/JAD-121264 23168450

[B4] Ansari U. Chen V. Sedighi R. Syed B. Muttalib Z. Ansari K.. (2023). Role of the UNC13 family in human diseases: a literature review. AIMS Neurosci. 10, 388–400. 10.3934/Neuroscience.2023029 38188011 PMC10767061

[B5] Aries M. L. Hensley-Mcbain T. (2023). Neutrophils as a potential therapeutic target in Alzheimer's disease. Front. Immunol. 14:8. 10.3389/fimmu.2023.1123149 36936930 PMC10020508

[B6] Bathini P. Brai E. Auber L A. (2019). Olfactory dysfunction in the pathophysiological continuum of dementia. Ageing Res. Rev. 55:100956. 10.1016/j.arr.2019.100956 31479764

[B7] Bergström S. Remnestål J. Yousef J. Olofsson J. Markaki I. Carvalho S.. (2021). Multi-cohort profiling reveals elevated CSF levels of brain-enriched proteins in Alzheimer's disease. Ann. Clin. Transl. Neur. 8, 1456–1470 10.1002/acn3.51402 34129723 PMC8283172

[B8] Bienenstock J. Kunze W. A. Forsythe P. (2018). Disruptive physiology: olfaction and the microbiome-gut-brain axis. Biol. Rev. 93, 390–403. 10.1111/brv.12348 28675687

[B9] Blacher E. Dadali T. Bespalko A. Haupenthal V J. Grimm M. O. Hartmann T.. (2015). Alzheimer's disease pathology is attenuated in a CD 38-deficient mouse model. Ann. Neurol. 78, 88–103. 10.1002/ana.24425 25893674 PMC6929692

[B10] Bui T. P. N. Mannerås-Holm L. Puschmann R. Wu H. Troise A D. Nijsse B.. (2021). Conversion of dietary inositol into propionate and acetate by commensal Anaerostipes associates with host health. Nat. Commun. 12:4798. 10.1038/s41467-021-25081-w 34376656 PMC8355322

[B11] Chen H. Meng L. Shen L. (2022). Multiple roles of short-chain fatty acids in Alzheimer disease. Nutrition 93:111499. 10.1016/j.nut.2021.111499 34735921

[B12] Chen Q. Q. Wu J. P. Dong X. X. Yin H. J. Shi X. F. Su S. Y.. (2021). Gut flora-targeted photobiomodulation therapy improves senile dementia in an Aβ-induced Alzheimer's disease animal model. J. Photochem. Photobiol. B-Biol. 216:12. 10.1016/j.jphotobiol.2021.112152 33610085

[B13] Chen Y-N. Kostka J. K. Bitzenhofer S. H. Hanganu-Opatz I L. (2023). Olfactory bulb activity shapes the development of entorhinal-hippocampal coupling and associated cognitive abilities. Curr. Biol. 33, 4353–4366.e4355. 10.1016/j.cub.2023.08.072 37729915 PMC10617757

[B14] Chettih S. N. Mackevicius E. L. Hale S. Aronov D. (2024). Barcoding of episodic memories in the hippocampus of a food-caching bird. Cell 187, 1922–1935.e1920. 10.1016/j.cell.2024.02.032 38554707 PMC11015962

[B15] Colombo A. V. Sadler R. K. Llovera G. Singh V. Roth S. Heindl S.. (2021). Microbiota-derived short chain fatty acids modulate microglia and promote Aβ plaque deposition. Elife 10:e59826. 10.7554/eLife.59826.sa2 33845942 PMC8043748

[B16] Connor E. E. Zhou Y. Liu G. E. (2018). The essence of appetite: does olfactory receptor variation play a role? J. Anim. Sci. 96, 1551–1558. 10.1093/jas/sky068 29534194 PMC6140904

[B17] Dahmani L. Patel R M. Yang Y. Chakravarty M M. Fellows L K. Bohbot V D. (2018). An intrinsic association between olfactory identification and spatial memory in humans. Nat. Commun. 9:4162. 10.1038/s41467-018-06569-4 30327469 PMC6191417

[B18] Dalile B. Van Oudenhove L. Vervliet B. Verbeke K. (2019). The role of short-chain fatty acids in microbiota–gut–brain communication. Nat. Rev. Gastroenterol. Hepatol. 16, 461–478 10.1038/s41575-019-0157-3 31123355

[B19] Devanand D. P. (2016). Olfactory identification deficits, cognitive decline, and dementia in older adults. Am. J. Geriatr. Psychiatr. 24, 1151–1157. 10.1016/j.jagp.2016.08.010 27745824 PMC5136312

[B20] Do Carmo S. Crynen G. Paradis T. Reed J. Iulita M F. Ducatenzeiler A.. (2018). Hippocampal proteomic analysis reveals distinct pathway deregulation profiles at early and late stages in a rat model of Alzheimer's-Like amyloid pathology. Mol. Neurobiol. 55, 3451–3476. 10.1007/s12035-017-0580-9 28502044

[B21] Doty R. L. (2008). The olfactory vector hypothesis of neurodegenerative disease: is it viable? Ann. Neurol. 63, 7–15. 10.1002/ana.21327 18232016

[B22] Doty R. L. (2021). The mechanisms of smell loss after SARS-CoV-2 infection. Lancet Neurol. 20, 693–695. 10.1016/S1474-4422(21)00202-7 34339627 PMC8324112

[B23] Doty R. L. Singh A. Tetrud J. Langston J. W. (1992). Lack of major olfactory dysfunction in MPTP-induced parkinsonism. Ann. Neurol. 32, 97–100. 10.1002/ana.410320116 1642478

[B24] Du Y. G. Li X. Y. An Y. Song Y. Lu Y. H. (2022). Association of gut microbiota with sort-chain fatty acids and inflammatory cytokines in diabetic patients with cognitive impairment: a cross-sectional, non-controlled study. Front. Nutr. 9:14. 10.3389/fnut.2022.930626 35938126 PMC9355148

[B25] Egorova P. Popugaeva E. Bezprozvanny I. (2015). Disturbed calcium signaling in spinocerebellar ataxias and Alzheimer's disease. Semin. Cell Dev. Biol. 40, 127–133. 10.1016/j.semcdb.2015.03.010 25846864 PMC4433580

[B26] Ekström I. A. Rizzuto D. Grande G. Bellander T. Laukka E. J. (2022). Environmental air pollution and olfactory decline in aging. Environ. Health. Persp. 130:027005. 10.1289/EHP9563 35139319 PMC8828267

[B27] Fan X. J. Zhang Y. Y. Song Y. Zhao Y. Y. Xu Y. A. Guo F.. (2023). Compound Danshen Dripping Pills moderate intestinal flora and the TLR4/MyD88/NF-κB signaling pathway in alleviating cognitive dysfunction in type 2 diabetic KK-Ay mice. Phytomedicine 111:17. 10.1016/j.phymed.2023.154656 36682300

[B28] Feng E. P. Yang X. F. Zhao K. M. Li Y. Zhu H. Y. Wang Z. S.. (2023). Gut microbiota is associated with spatial memory and seed-hoarding behavior of South China field mice (*Apodemus draco*). *Front. Microbiol*. 14:10. 10.3389/fmicb.2023.1236359 37771706 PMC10525317

[B29] Fjaeldstad A. W. Smith B. (2022). The effects of olfactory loss and parosmia on food and cooking habits, sensory awareness, and quality of life-a possible avenue for regaining enjoyment of food. Foods 11:16. 10.3390/foods11121686 35741884 PMC9222253

[B30] Forsythe P. Bienenstock J. Kunze W. A (2014). Microbial Endocrinology: The Microbiota-Gut-Brain Axis in Health and Disease, eds. Lyte, M. and Cryan, J.F. (New York: Springer), 115–133. 10.1007/978-1-4939-0897-4_5

[B31] Gao C. Li B. Y. He Y. X. Huang P. Du J. J. He G. Y.. (2023a). Early changes of fecal short-chain fatty acid levels in patients with mild cognitive impairments. CNS Neurosci. Ther. 29, 3657–3666. 10.1111/cns.14252 37144597 PMC10580335

[B32] Gao H. Zhang Y, X. Luo D, L. Xu J. Tan S. W. Li Y.. (2023b). Activation of the hippocampal DRD2 alleviates neuroinflammation, synaptic plasticity damage and cognitive impairment after sleep deprivation. Mol. Neurobiol. 60, 7208–7221. 10.1007/s12035-023-03514-5 37543530

[B33] Gerkin R. C. Ohla K. Veldhuizen M. G. Joseph P. V. Kelly C. E. Bakke A. J.. (2021). Recent smell loss is the best predictor of COVID-19 among individuals with recent respiratory symptoms. Chem Senses 46:12. 10.1093/chemse/bjaa081 33367502 PMC7799216

[B34] Giil L. M. Midttun Ø. Refsum H. Ulvik A. Advani R. Smith A D.. (2017). Kynurenine pathway metabolites in Alzheimer's disease. J Alzheimer's Dis, 60, 495–504. 10.3233/JAD-170485 28869479

[B35] Gillette-Guyonnet S. Nourhashemi F. Andrieu S. De Glisezinski I. Ousset P J. Riviere D.. (2000). Weight loss in Alzheimer disease. Am. J. Clin. Nut. 71, 637S-642S. 10.1093/ajcn/71.2.637s 10681272

[B36] Guadalupe-Fernandez V. De Sario M. Vecchi S. Bauleo L. Michelozzi P. Davoli M.. (2021). Industrial odour pollution and human health: a systematic review and meta-analysis. Environ. Health 20, 1–21. 10.1186/s12940-021-00774-3 34551760 PMC8459501

[B37] Guerreiro S. Privat A-L Bressac L. Toulorge D. (2020). CD38 in neurodegeneration and neuroinflammation. Cells 9:471. 10.3390/cells9020471 32085567 PMC7072759

[B38] Harel D. Carmel L. Lancet D. (2003). Towards an odor communication system. Comput. Biol. Chem. 27, 121–133. 10.1016/S1476-9271(02)00092-0 12821309

[B39] Hintze L. J. Goldfield G. Seguin R. Damphousse A. Riopel A. Doucet É. (2019). The rate of weight loss does not affect resting energy expenditure and appetite sensations differently in women living with overweight and obesity. Physiol. Behav. 199, 314–321. 10.1016/j.physbeh.2018.11.032 30496740

[B40] Iqbal T. Byrd-Jacobs C. (2010). Rapid degeneration and regeneration of the zebrafish olfactory epithelium after Triton X-100 application. Chem. Senses 35, 351–361. 10.1093/chemse/bjq019 20228140 PMC2871777

[B41] Kielar C. Sawiak S J. Navarro Negredo P. Tse D H. Morton A J. (2012). Tensor-based morphometry and stereology reveal brain pathology in the complexin1 knockout mouse. PLoS ONE 7:e32636. 10.1371/journal.pone.0032636 22393426 PMC3290572

[B42] Kim B, Y. Bae J. H. (2022). Olfactory function and depression: a meta-analysis. Ear Nose Throat J. 8. 10.1177/01455613211056553 35360974

[B43] Leipnitz G. Schumacher C. Dalcin K B. Scussiato K. Solano A. Funchal C.. (2007). *In vitro* evidence for an antioxidant role of 3-hydroxykynurenine and 3-hydroxyanthranilic acid in the brain. Neurochem. Int. 50, 83–94. 10.1016/j.neuint.2006.04.017 16959377

[B44] Li Y. L Ning L. Yin Y. R. Wang R. Zhang Z. Y. Hao L. J.. (2020). Age-related shifts in gut microbiota contribute to cognitive decline in aged rats. Aging-Us, 12, 7801–7817. 10.18632/aging.103093 32357144 PMC7244050

[B45] Li J. Liao X J. Yin X. D. Deng Z. M. Hu G. F. Zhang W. W.. (2023). Gut microbiome and serum metabolome profiles of capsaicin with cognitive benefits in APP/PS1 Mice. Nutrients 15:16. 10.3390/nu15010118 36615777 PMC9823564

[B46] Li L. Su Y. Li F. Wang Y. Ma Z. Li Z.. (2020). The effects of daily fasting hours on shaping gut microbiota in mice. BMC Microbiol. 20, 1–8. 10.1186/s12866-020-01754-2 32209070 PMC7092480

[B47] Li X. Zhao Y. Xia Q. Zheng L. Liu L. Zhao B.. (2016). Nuclear translocation of annexin 1 following oxygen-glucose deprivation–reperfusion induces apoptosis by regulating Bid expression via p53 binding. Cell Death Dis. 7, e2356–e2356. 10.1038/cddis.2016.259 27584794 PMC5059862

[B48] Liao K. Liu D. Zhu L. Q. (2012). Enriched odor exposure decrease tau phosphorylation in the rat hippocampus and cortex. Neurosci. Lett. 507, 22–26. 10.1016/j.neulet.2011.11.040 22155098

[B49] Lyte M. (2014). Microbial endocrinology host-microbiota neuroendocrine interactions influencing brain and behavior. Gut Microbes 5, 381–389. 10.4161/gmic.28682 24690573 PMC4153777

[B50] Maierhaba X. Y. Mahmut M. Yao Y. Jia Y. (2018). iTRAQ-based proteomics analysis of hippocampus and striatum in rats with hyperglycemia and Parkinson's disease. Int. J. Clin. Exp. Med. 11, 1959–1972.

[B51] Mango D. Saidi A. Cisale G Y. Feligioni M. Corbo M. Nisticò R. (2019). Targeting synaptic plasticity in experimental models of Alzheimer's disease. Front. Pharmacol. 10:8. 10.3389/fphar.2019.00778 31379566 PMC6646937

[B52] Martins I. J. Fernando W. M. (2014). High fibre diets and Alzheimer's disease. Food Nutrition Sci. 5, 410–424. 10.4236/fns.2014.54049

[B53] Maurer M, L. Goyco-Blas J, F. Kohl K, D. (2024). Dietary tannins alter growth, behavior, and the gut microbiome of larval amphibians. Integr. Zool. 19, 585–595. 10.1111/1749-4877.12758 37551631

[B54] Mitrea L. Nemeş S-A. Szabo K. Teleky B-E. Vodnar D-C. (2022). Guts imbalance imbalances the brain: a review of gut microbiota association with neurological and psychiatric disorders. Front. Med. 9:706. 10.3389/fmed.2022.813204 35433746 PMC9009523

[B55] Moftakhar P. Lynch M D. Pomakian J L. Vinters H V. (2010). Aquaporin expression in the brains of patients with or without cerebral amyloid angiopathy. J Neuropath. Exp. Neur. 69, 1201–1209. 10.1097/NEN.0b013e3181fd252c 21107133 PMC3155418

[B56] Nagpal R. Neth B J. Wang S. Craft S. Yadav H. (2019). Modified Mediterranean-ketogenic diet modulates gut microbiome and short-chain fatty acids in association with Alzheimer's disease markers in subjects with mild cognitive impairment. EBioMedicine 47, 529–542. 10.1016/j.ebiom.2019.08.032 31477562 PMC6796564

[B57] Naudon L. François A. Mariadassou M. Monnoye M. Philippe C. Bruneau A.. (2020). First step of odorant detection in the olfactory epithelium and olfactory preferences differ according to the microbiota profile in mice. Behav. Brain Res. 384:9. 10.1016/j.bbr.2020.112549 32050097

[B58] Nielsen J. E. Maltesen R. G. Havelund J. F. Fargeman N. J. Gotfredsen C. H. Vestergard K.. (2021). Characterising Alzheimer's disease through integrative NMR- and LC-MS-based metabolomics. Metabolism Open 12:100125. 10.1016/j.metop.2021.100125 34622190 PMC8479251

[B59] Ogawa M. Fukuyama H. Ouchi Y. Yamauchi H. Kimura J. (1996). Altered energy metabolism in Alzheimer's disease. J. Neurol. Sci. 139, 78–82. 10.1016/0022-510X(96)00033-0 8836976

[B60] Ohland C. L. Kish L. Bell H. Thiesen A. Hotte N. Pankiv E.. (2013). Effects of Lactobacillus helveticus on murine behavior are dependent on diet and genotype and correlate with alterations in the gut microbiome. Psychoneuroendocrinology 38, 1738–1747. 10.1016/j.psyneuen.2013.02.008 23566632

[B61] Paskin T. R. Byrd-Jacobs C. A. (2012). Reversible deafferentation of the adult zebrafish olfactory bulb affects glomerular distribution and olfactory-mediated behavior. Behav. Brain Res. 235, 293–301. 10.1016/j.bbr.2012.08.018 22963994 PMC3445742

[B62] Pieniak M. Oleszkiewicz A. Avaro V. Calegari F. Hummel T. (2022). Olfactory training - Thirteen years of research reviewed. Neurosci. Biobehav. Rev. 141:19. 10.1016/j.neubiorev.2022.104853 36064146

[B63] Plagman A. Hoscheidt S. Mclimans K. E Klinedinst B. Pappas C. Anantharam V.. (2019). Cholecystokinin and Alzheimer's disease: a biomarker of metabolic function, neural integrity, and cognitive performance. Neurobiol. Aging 76, 201–207. 10.1016/j.neurobiolaging.2019.01.002 30739077 PMC6425756

[B64] Prediger R. D. S. Batista L C. Medeiros R. Pandolfo P. Florio J C. Takahashi R N. (2006). The risk is in the air: Intranasal administration of MPTP to rats reproducing clinical features of Parkinson's disease. Exp. Neurol. 202, 391–403. 10.1016/j.expneurol.2006.07.001 16908021

[B65] Rathore K. Shukla N. Naik S. Sambhav K. Dange K. Bhuyan D.. (2025). The bidirectional relationship between the gut microbiome and mental health: a comprehensive review. Cureus 17:e80810. 10.7759/cureus.80810 40255763 PMC12007925

[B66] Reinikainen K. Soininen H. Riekkinen P. (1990). Neurotransmitter changes in Alzheimer's disease: implications to diagnostics and therapy. J. Neurosci. Res. 27, 576–586. 10.1002/jnr.490270419 1981917

[B67] Rusznak Z. Sengul G. Paxinos G. Kim W, S. Fu Y, H. (2018). Odor enrichment increases hippocampal neuron numbers in mouse. Exp. Neurobiol. 27, 94–102. 10.5607/en.2018.27.2.94 29731675 PMC5934547

[B68] Saha S. Hatch D. J. Hayden K. M. Steffens D. C. Potter G. G. (2016). Appetite and weight loss symptoms in late-life depression predict dementia outcomes. Am. J. Geriatr. Psychiatr. 24, 870–878. 10.1016/j.jagp.2016.05.004 27555110 PMC6473118

[B69] Salimi M. Tabasi F. Nazari M. Ghazvineh S. Raoufy M. R. (2022). The olfactory bulb coordinates the ventral hippocampus–medial prefrontal cortex circuit during spatial working memory performance. J. Physiol. Sci. 72:9. 10.1186/s12576-022-00833-5 35468718 PMC10717655

[B70] Sando R. Gounko N. Pieraut S. Liao L J. Yates J. Maximov A. (2012). HDAC4 governs a transcriptional program essential for synaptic plasticity and memory. Cell 151, 821–834. 10.1016/j.cell.2012.09.037 23141539 PMC3496186

[B71] Sarafoleanu C. Mella C. Georgescu M. Perederco C. (2009). The importance of the olfactory sense in the human behavior and evolution. J. Med. Life 2:196. 20108540 PMC3018978

[B72] Saura C. A. Deprada A. Capilla-López M. D. Parra-Damas A. (2023). Revealing cell vulnerability in Alzheimer's disease by single-cell transcriptomics. Semin. Cell Dev. Biol. 139, 73–83. 10.1016/j.semcdb.2022.05.007 35623983

[B73] Shafqat A. Noor Eddin A. Adi G. Al-Rimawi M. Abdul Rab S. Abu-Shaar M.. (2023). Neutrophil extracellular traps in central nervous system pathologies: a mini review. Front. Med. 10:1083242. 10.3389/fmed.2023.1083242 36873885 PMC9981681

[B74] Sharma A. Kumar R. Aier I. Semwal R. Tyagi P. Varadwaj P. (2019). Sense of smell: structural, functional, mechanistic advancements and challenges in human olfactory research. Curr. Neuropharmacol. 17, 891–911. 10.2174/1570159X17666181206095626 30520376 PMC7052838

[B75] Sharma V. K. Singh T, G. Singh S. Garg N. Dhiman S. (2021). Apoptotic pathways and Alzheimer's disease: probing therapeutic potential. Neurochem. Res. 46, 3103–3122. 10.1007/s11064-021-03418-7 34386919

[B76] Shigeo T. Jeong Seop R. Christian R. Reinhard J. (2001). Identification of differentiation-associated brain-specific phosphate transporter as a second vesicular glutamate transporter (VGLUT2). J. Neurosci. 21:RC182. 10.1523/JNEUROSCI.21-22-j0002.2001 11698620 PMC6762262

[B77] Snowden S. G. Ebshiana A. A. Hye A. Pletnikova O. O'brien R. Yang A.. (2019). Neurotransmitter imbalance in the brain and Alzheimer's disease pathology. J. Alzheimer's Dis. 72, 35–43. 10.3233/JAD-190577 31561368 PMC12961646

[B78] Stevenson R. J. (2010). An initial evaluation of the functions of human olfaction. Chem. Senses 35, 3–20. 10.1093/chemse/bjp083 19942579

[B79] Sushma and Mondal, A. C.. (2019). Role of GPCR signaling and calcium dysregulation in Alzheimer's disease. Mol. Cell. Neurosci. 101:11. 10.1016/j.mcn.2019.103414 31655116

[B80] Teker H. T. Ceylani T. (2023). Intermittent fasting supports the balance of the gut microbiota composition. Int. Microbiol. 26, 51–57. 10.1007/s10123-022-00272-7 35953616

[B81] Trillaud-Doppia E. Paradis-Isler N. Boehm J. (2016). A single amino acid difference between the intracellular domains of amyloid precursor protein and amyloid-like precursor protein 2 enables induction of synaptic depression and block of long-term potentiation. Neurobiol. Dis. 91, 94–104 10.1016/j.nbd.2016.02.016 26921470

[B82] Vance D. E. Del Bene V. A. Kamath V. Frank J, S. Billings R. Cho D. Y.. (2024). Does olfactory training improve brain function and cognition? A systematic review. Neuropsychol. Rev. 34, 155–191. 10.1007/s11065-022-09573-0 36725781 PMC9891899

[B83] Wanapaisan P. Chuansangeam M. Nopnipa S. Mathuranyanon R. Nonthabenjawan N. Ngamsombat C.. (2023). Association between gut microbiota with mild cognitive impairment and Alzheimer's disease in a Thai population. Neurodegener. Dis. 22, 43–54. 10.1159/000526947 36070704

[B84] Wang C. F. Zhao C. C. He Y. Li Z. Y. Liu W. L. Huang X. J.. (2019). Mild hypothermia reduces endoplasmic reticulum stress-induced apoptosis and improves neuronal functions after severe traumatic brain injury. Brain Behav. 9:e01248. 10.1002/brb3.1248 30834702 PMC6456779

[B85] Wang H-X. Wang Y-P. (2016). Gut microbiota-brain axis. Chin. Med. J. 129, 2373–2380. 10.4103/0366-6999.190667 27647198 PMC5040025

[B86] Wang Y. He X. N. Qian Z. J. Li S. X. Jing M. Z. Li X. X.. (2025). Exploring dietary composition in an invasive apple snail from different habitats combining with intestinal microbiota and metabolomics. Integr. Zool. 10.1111/1749-4877.12942. [Epub ahead of print]. 39794878

[B87] Wang Z. Chen W-H. Li S-X. He Z-M. Zhu W-L. Ji Y-B.. (2021). Gut microbiota modulates the inflammatory response and cognitive impairment induced by sleep deprivation. Mol. Physiol. 26, 6277–6292. 10.1038/s41380-021-01113-1 33963281

[B88] Watase K. Gatchel J. R. Sun Y. Emamian E. Atkinson R. Richman R.. (2007). Lithium therapy improves neurological function and hippocampal dendritic arborization in a spinocerebellar ataxia type 1 mouse model. Plos Med. 4:e182. 10.1371/journal.pmed.0040182 17535104 PMC1880853

[B89] Wu L. Han Y. Zheng Z. Peng G. Liu P. Yue S.. (2021). Altered gut microbial metabolites in amnestic mild cognitive impairment and Alzheimer's disease: signals in host–microbe interplay. Nutrients 13:228. 10.3390/nu13010228 33466861 PMC7829997

[B90] Wu Z. Liu H. Yan E. Zhang X. Wang Y. Huang C.. (2023). Desulfovibrio confers resilience to the comorbidity of pain and anxiety in a mouse model of chronic inflammatory pain. Psychopharmacology 240, 87–100. 10.1007/s00213-022-06277-4 36441221

[B91] Xu Y. Wei S. Zhu L. Huang C. Yang T. Wang S.. (2023). Low expression of the intestinal metabolite butyric acid and the corresponding memory pattern regulate HDAC4 to promote apoptosis in rat hippocampal neurons. Ecotox Environ Safe 253:114660. 10.1016/j.ecoenv.2023.114660 36812872

[B92] Yan Y. Aierken A. Wang C. J. Song D. Ni J. J. Wang Z.. (2022). A potential biomarker of preclinical Alzheimer's disease: The olfactory dysfunction and its pathogenesis-based neural circuitry impairments. Neurosci. Biobehav. Rev. 132, 857–869. 10.1016/j.neubiorev.2021.11.009 34810025

[B93] Yang Y. H. Lu M. M. Xu Y. C. Qian J. Le G, W. Xie Y L. (2022). Dietary methionine via dose-dependent inhibition of short-chain fatty acid production capacity contributed to a potential risk of cognitive dysfunction in mice. J. Agric. Food Chem. 70, 15225–15243. 10.1021/acs.jafc.2c04847 36413479

[B94] Yang Z. Zou Y. Wang L. (2023). Neurotransmitters in prevention and treatment of Alzheimer's disease. Int. J. Mol. Sci. 24:3841. 10.3390/ijms24043841 36835251 PMC9966535

[B95] Yeomans M. R. (2006). Olfactory influences on appetite and satiety in humans. Physiol. Behav. 87, 800–804. 10.1016/j.physbeh.2006.01.029 16545846

[B96] Yi X. Cha M. (2022). Gut Dysbiosis has the potential to reduce the sexual attractiveness of mouse female. Front. Microbiol. 13:916766. 10.3389/fmicb.2022.916766 35677910 PMC9169628

[B97] Yi X. F. Yi S. J. Deng Y. H. Wang M. H. Ju M. Y. (2021). High-valued seeds are remembered better: evidence for item-based spatial memory of scatter-hoarding rodents. Anim. Behav. 175, 1–6. 10.1016/j.anbehav.2021.02.009

[B98] Zenaro E. Pietronigro E. Della Bianca V. Piacentino G. Marongiu L. Budui S.. (2015). Neutrophils promote Alzheimer's disease-like pathology and cognitive decline via LFA-1 integrin. Nat. Med. 21, 880–886. 10.1038/nm.3913 26214837

[B99] Zeppenfeld D. M. Simon M. Haswell J. D. D'abreo D. Murchison C. Quinn J, F.. (2017). Association of perivascular localization of aquaporin-4 with cognition and Alzheimer disease in aging brains. JAMA Neurol. 74, 91–99. 10.1001/jamaneurol.2016.4370 27893874

[B100] Zhao H. Zhou X. Song Y. Zhao W. Sun Z. Zhu J.. (2025). Multi-omics analyses identify gut microbiota-fecal metabolites-brain-cognition pathways in the Alzheimer's disease continuum. Alzheimer's Res. Therapy 17:36. 10.1186/s13195-025-01683-0 39893498 PMC11786436

[B101] Zhao X. Guo J. Wang Y. Yi X. (2024). High-tannin food enhances spatial memory and scatter-hoarding in rodents via the microbiota-gut-brain axis. Microbiome 12:140. 10.1186/s40168-024-01849-2 39075602 PMC11285206

[B102] Zhou J. Wang M. H. Yi X. F. (2022). Alteration of gut microbiota of a food-storing hibernator, Siberian Chipmunk *Tamias sibiricus*. Microb. Ecol. 84, 603–612. 10.1007/s00248-021-01877-7 34562129

[B103] Zilberter Y. Zilberter M. (2017). The vicious circle of hypometabolism in neurodegenerative diseases: ways and mechanisms of metabolic correction. J. Neurosci. Res. 95, 2217–2235. 10.1002/jnr.24064 28463438

