# Supplementary material for: The potential of olfaction loss to induce cognitive impairment and anxiety behavior in mice via the microbiota-gut-brain axis
Source: Front Microbiol. 2025 Jul 2;16:1595742. doi: 10.3389/fmicb.2025.1595742 (PMC12265083; doi:10.3389/fmicb.2025.1595742)

Supplementary figures

Figure S1

Gut microbiota analysis of the control and anosmic groups. Alpha rarefaction plot of PD whole tree and ACE (A, B); Rank Abundance analysis curve (C); and Specaccum species accumulation curve (D).

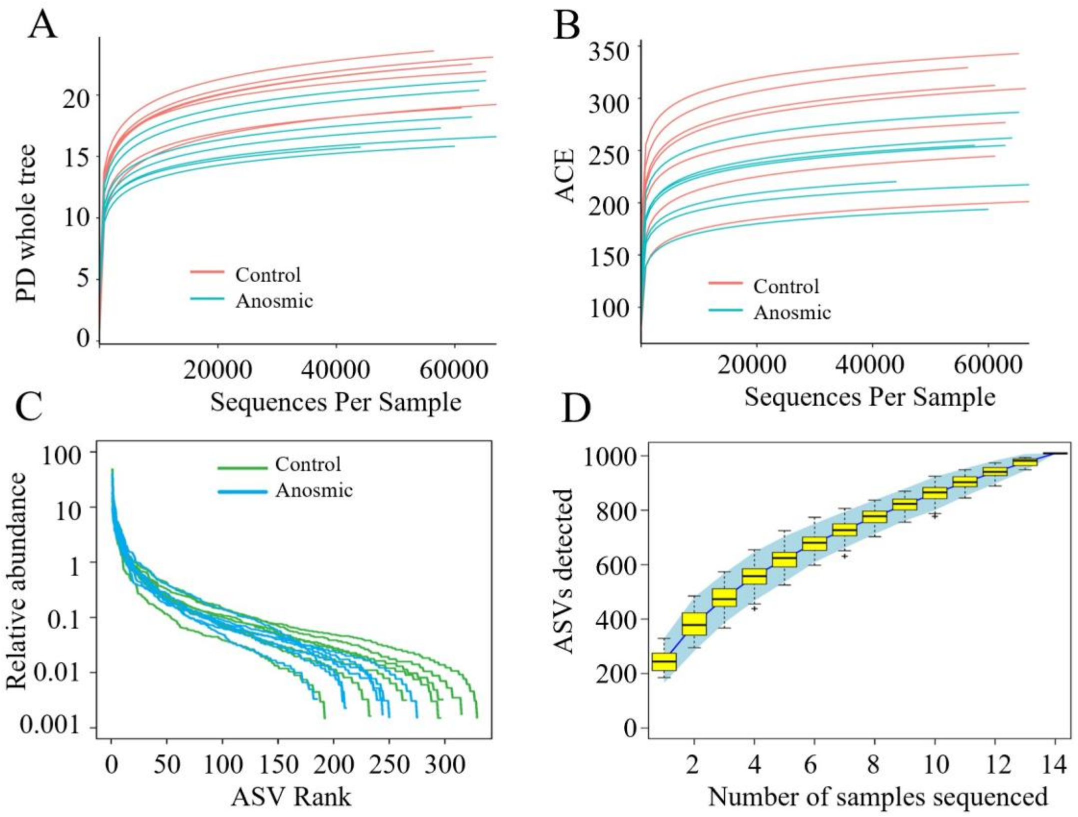

Figure S2

PCoA based on the Bray–Curtis distance matrix of the mouse gut microbiota. Control and anosmic groups (A); A-FMT and C-FMT groups (B).

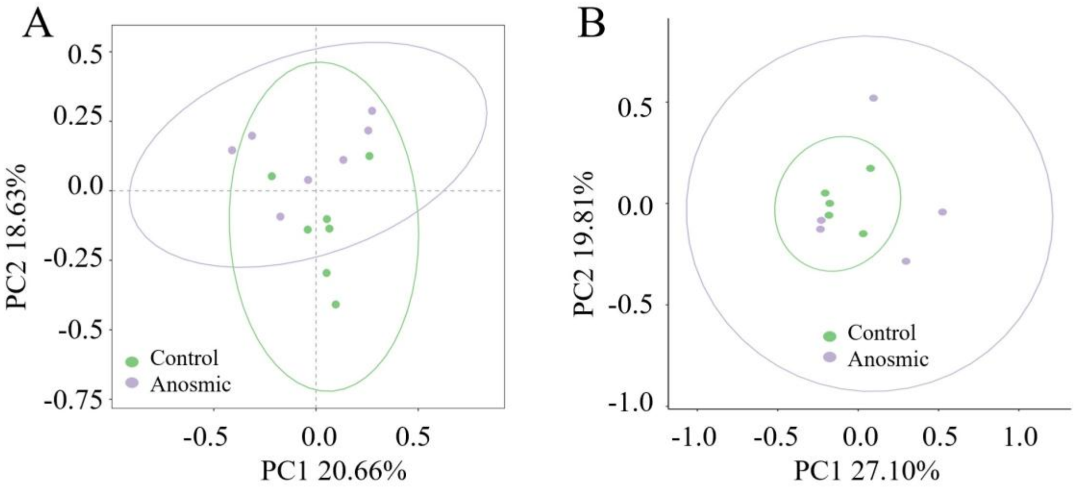

Figure S3

Pep-Quant library characteristics of olfactory bulbs. Bar graph showing distribution of identified peptides and proteins (A), molecular weight of proteins (B), protein length (C), proteome sequence coverage (D), peptide charge (E), and Pearson correlation coefficient (F).

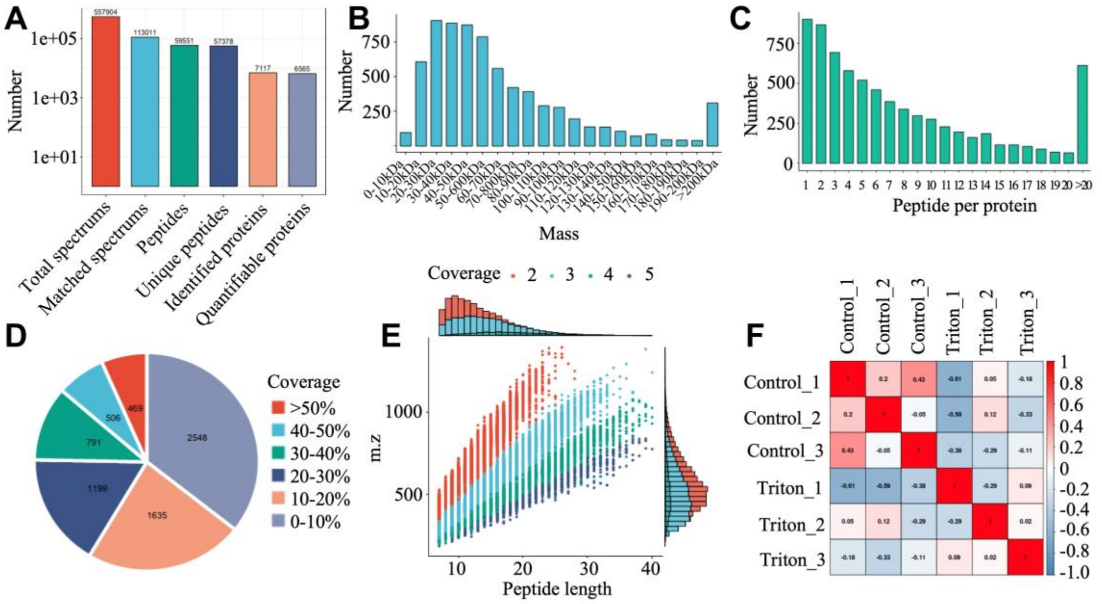

Figure S4

Pep-Quant library characteristics of mouse hippocampus. Bar graph showing distribution of identified peptides and proteins (A), molecular weight of proteins (B), protein length (C), proteome sequence coverage (D), peptide charge (E), and Pearson correlation coefficient (F).

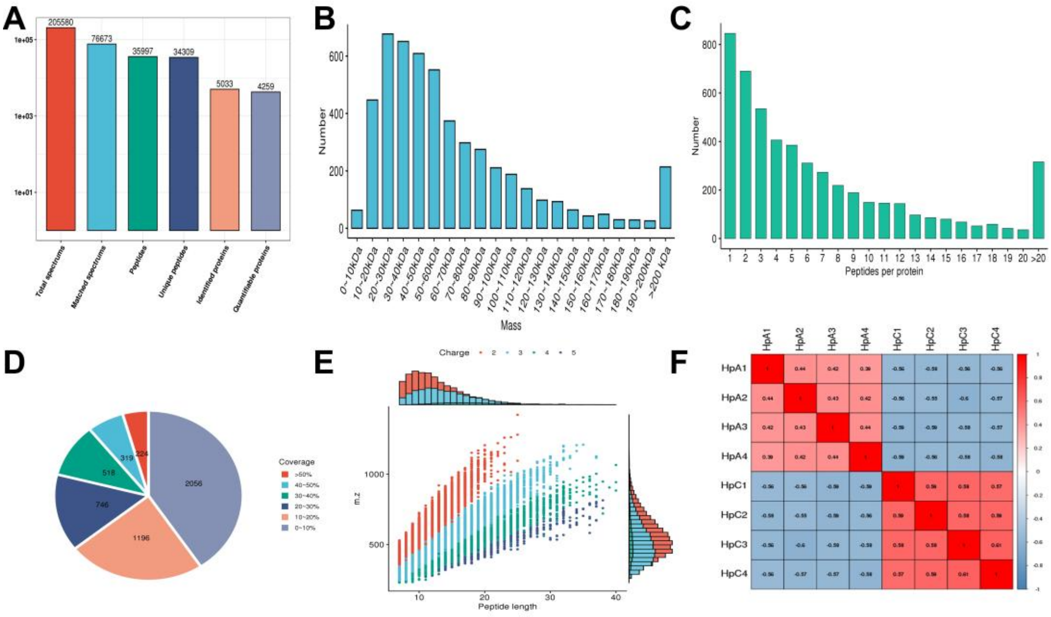

Figure S5. Schematic diagram of the KEGG pathways of the olfactory bulb proteome

Necroptosis signaling pathway (A) and synaptic vesicle cycle signaling pathway (B).

A

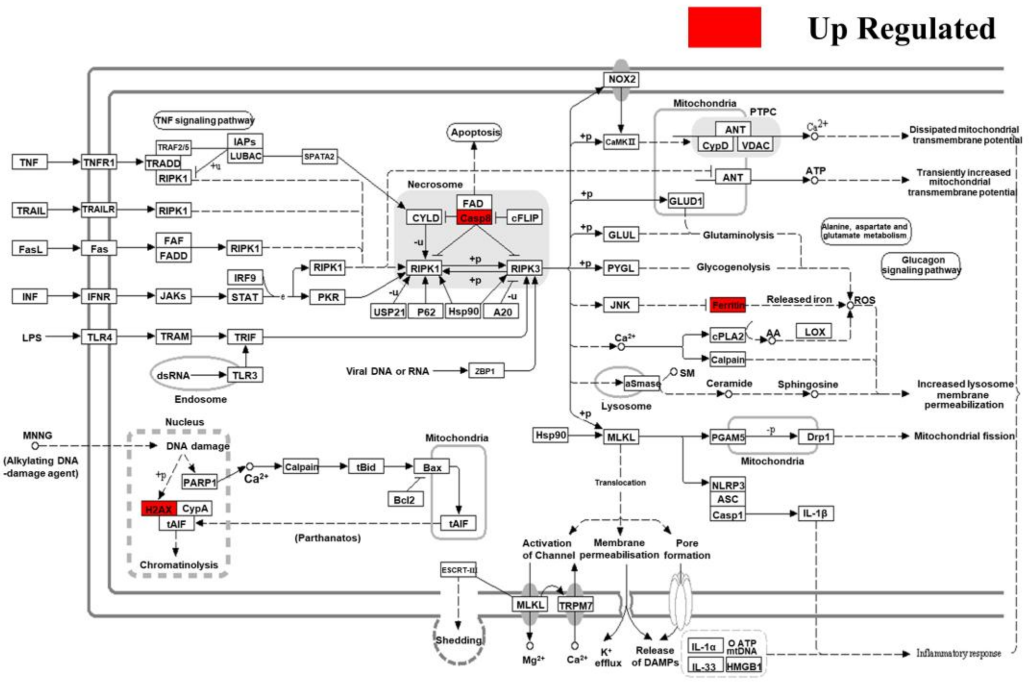

B

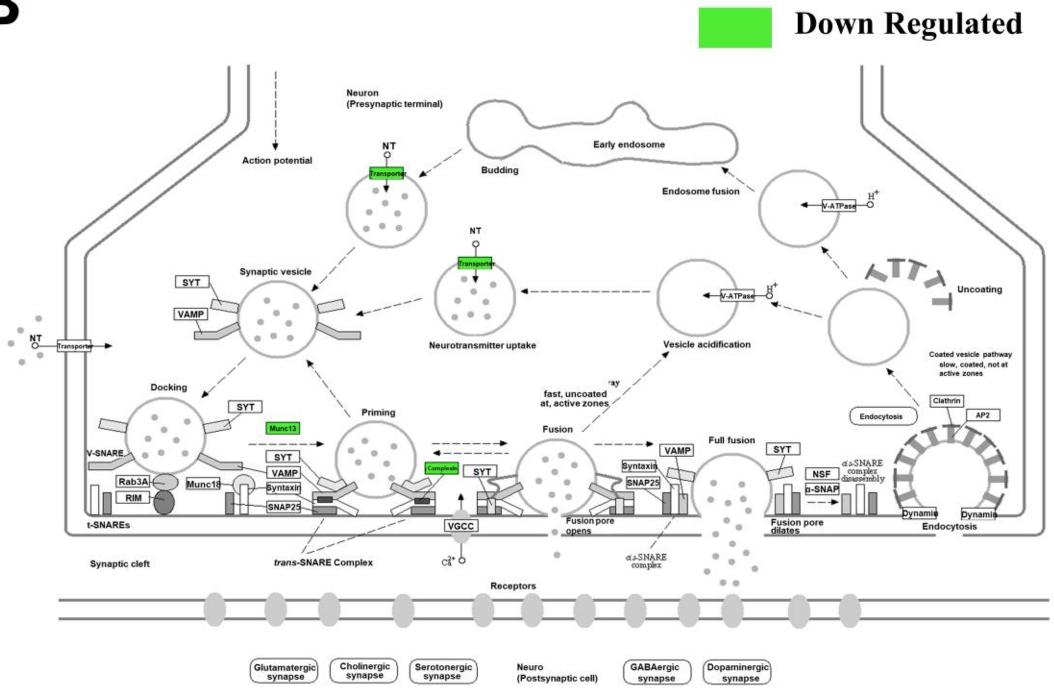

Figure S6. Schematic diagram of the KEGG pathways in the hippocampal proteome

Calcium signaling pathway (A), long-term depression pathway (B), neutrophil extracellular trap formation pathway (C), and spinocerebellar ataxia pathway (D).

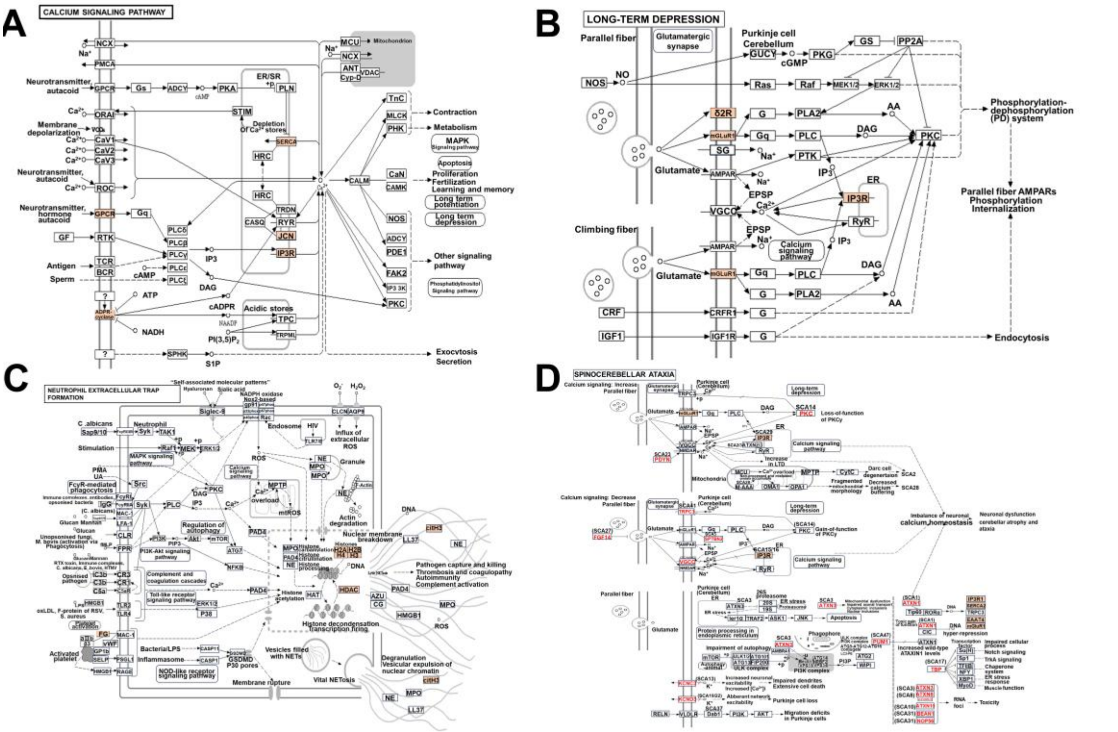

Figure S7. Correlation analyses between differential proteins in the hippocampus and BNS maze

Correlation analyses of AQP4, FGG, CD38, HDAC4, and COL1A1 with BNS maze experiment (time spent in the target zone) (Figure S7A). Correlation analyses of CCK, PLP1, and CRYAB with BNS maze experiment (time spent in the target zone) (Figure S7B).

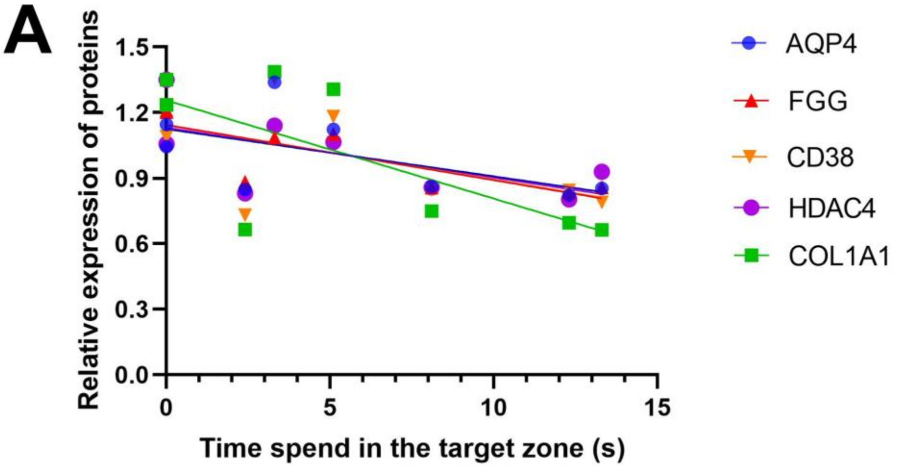

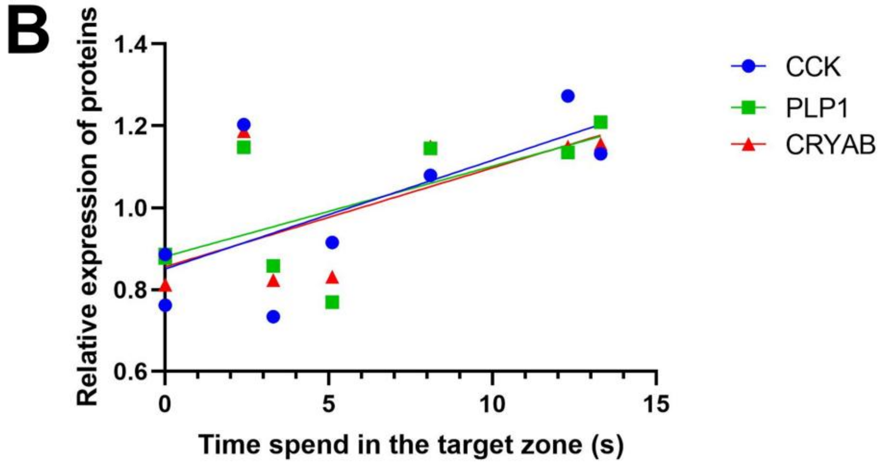

Supplement: Supplementary file 1 [file Data_Sheet_1.pdf]
